# Myofascial Pain Syndrome in Women with Primary Dysmenorrhea: A Case-Control Study

**DOI:** 10.3390/diagnostics12112723

**Published:** 2022-11-07

**Authors:** Ana Serrano-Imedio, Cesar Calvo-Lobo, Coraima Casañas-Martin, Alejandro Garrido-Marin, Daniel Pecos-Martin

**Affiliations:** 1 Grupo de Investigación en Fisioterapia y Dolor, Departamento de Fisioterapia, Facultad de Enfermería y Fisioterapia, Universidad de Alcalá, 28801 Alcalá de Henares, Spain; 2 CARMASALUD Clinical and Research Center, 28037 Madrid, Spain; 3 Facultad de Enfermería, Fisioterapia y Podología, Universidad Complutense de Madrid, 28040 Madrid, Spain; 4 Department of Physiotherapy, Faculty of Biomedical and Health Sciences, Universidad Europea de Madrid, 28670 Madrid, Spain; 5 Medical Services, Getafe C.F., 28905 Getafe, Spain

**Keywords:** primary dysmenorrhea, myofascial pain syndrome, myofascial trigger points, mechanosensitivity, pain pressure threshold

## Abstract

There is limited information on myofascial trigger points (MTrPs) and specific symptoms of chronic pelvic pain and, more specifically, dysmenorrhea. The objective of this study was to determine whether patients suffering from primary dysmenorrhea present alterations in mechanosensitivity and pain patterns, and greater presence of MTrPs in the abdominal and pelvic floor muscles. A case-control study was carried out with a total sample of 84 participants distributed based on primary dysmenorrhea and contraceptive treatment. The sample was divided into four groups each comprising 21 women. Data on pain, quality of life, and productivity and work absenteeism were collected; three assessments were made in different phases of the menstrual cycle, to report data on pressure pain threshold, MTrP presence, and referred pain areas. One-way ANOVA tests showed statistically significant differences (*p* < 0.01) between the groups, for the Physical Health domain and the total score of the SF-12 questionnaire, and for all the domains of the McGill questionnaire; but no significant differences were found in the data from the WPAI-GH questionnaire. Statistically significant data (*p* < 0.01) were found for mechanosensitivity in the abdominal area and limbs, but not for the lumbar assessment, within the group, with very few significant intergroup differences. The frequency of active MTrPs is higher in the groups of women with primary dysmenorrhea and during the menstrual phase, with the prevalence of myofascial trigger points of the iliococcygeus muscle being especially high in all examination groups (>50%) and higher than 70% in women with primary dysmenorrhea, in the menstrual phase, and the internal obturator muscle (100%) in the menstrual phase. Referred pain areas of the pelvic floor muscles increase in women with primary dysmenorrhea.

## 1. Introduction

Dysmenorrhea is considered one of the most prevalent gynecological disorders in women of reproductive age [1,2], reaching prevalence rates of up to 97% of the population in some locations [3], and ranging in Spain from 61.9% to 84.7% of the population [4,5]. Dysmenorrhea can be classified as secondary dysmenorrhea when pathological causes of the uterus or pelvis are responsible for the pain, the most frequent being endometriosis and pelvic inflammatory disorders, in addition to other causes, such as adenomyosis, uterine fibroids (myomas) or polyps, ovarian cysts, uterine malformations, pelvic adhesions, cervical stenosis of the uterus, pelvic congestion syndrome, and irritable bowel syndrome [1,6,7,8]. Primary dysmenorrhea (PD) is determined by a set of symptoms that appears to be associated with menstruation in the absence of relevant organic pathology that explains it, and that presents pain described as moderate or severe and that is fundamentally associated with an increase in the production of prostaglandins (PGs) at the endometrial level [2]. The etiology of PD is characterized by an increase in the synthesis and release of PGs, which cause hypercontractability of the myometrium with respect to the normal dynamics of the menstrual uterus. Hypercontractibility causes uterine ischemia and hypoxia and, consequently, an accumulation of metabolites and acids, which are also responsible for pain [5,9,10,11,12]. This increased contractility is mediated by the local release of nociceptive substances in response to hormonal stimuli. There is also a sensitization of nerve endings to nociceptive stimuli [5]. The increased volume of menstrual flow due to ovulatory cycles increases the release of uterine PGs, which stimulates uterine contraction, increasing the tone, frequency, and intensity of contractions and causing colic-like pain [5]. However, Akman et al., found that in young women with PD, there are no differences in pain regardless of whether the cycles are ovulatory or anovulatory [13]. In addition, historically, menstrual pain was related to psychological factors [14]. However, these theories have lost credibility as biochemical knowledge has increased, assuming that psychological problems are more a consequence than a cause [15,16].

This condition of cyclical and chronic pain also means a decrease in both the quality of life of patients [17] and in their academic and/or professional development, presenting itself as the main cause of absenteeism [1,18]. Despite these data and the high prevalence of menstrual pain, underdiagnosis and the absence or inadequacy of treatment are prominent problems in the majority of cases [8]. Data indicate that only one in three women consults a health professional for menstrual pain, and the vast majority also consult pharmacists instead of doctors or nurses [19]. This could be one of the reasons why the most common forms of treatment are pharmacological; for example, with analgesics aimed at alleviating symptoms at the time of menstruation [10,19], or through contraceptives aimed at reducing endometrial activity throughout the menstrual cycle [20]. Despite presenting greater side-effects relative to more conservative forms of treatment, they continue to be used more frequently because they provide greater relief of symptoms [21].

Studies on conservative forms of treatment other than pharmaceuticals remain highly limited and heterogeneous, complicating the search for therapeutic alternatives. There have been studies examining physiotherapy as a treatment for the management of PD, and in recent years studies have aimed at treating MTrPs in this type of patient [22,23,24].

The myofascial component is considered one of the most common diagnoses in clinical practice for chronic pelvic pain (CPP) [25], including PD in the classification of the *International Association for the Study of Pain* (IASP) as a condition of CPP [26]. Focusing on myofascial pain syndrome (MPS) shows that it shares clinical characteristics with PD that extend beyond pain, such as the fact that it causes autonomic symptoms [27], emotional disorders [28], or changes in mood [29], in addition to considering the central sensitization associated with both [29,30].

The diagnosis of MTrPs has been widely debated and studied in recent years [31,32,33]. Diagnosis has been based on clinical history and physical examination by palpation, and following the widely extended criteria described in 1999 by Travell and Simons; these are the presence of a tender point within a palpable taut band in skeletal muscle, exhibiting a local spasm response to sudden palpation of this taut band, and causing referred pain in response to stimulation or compression of the MTrPs [34,35].

Both active MTrPs and latent MTrPs induce peripheral and even central sensitization mechanisms [35,36] and, therefore, are related to a decrease in pain pressure thresholds (PPTs) [37]. Another of the characteristics of MTrPs is referred pain [38], with the areas of representation of referred pain being the rectus abdominis, external oblique, internal oblique, adductor magnus, gluteal, and quadratus lumborum muscles, very similar to menstrual pain [7,8,9,27,39,40,41].

MTrPs can be present in any skeletal muscle, including the pelvic floor (PF), where they can refer pain to the urethra, vagina, rectum, coccyx, sacrum, lower back, lower abdomen, and posterior thighs, as well as muscles in other body locations such as the back or hip; all of these cause pain to the pelvic area [39,42]. MTrPs may also be responsible for other symptoms compatible with some gynecological, gastrointestinal, and urological disorders or conditions [42,43]. We found studies in which the presence of MTrPs in the abdominal and pelvic musculature may be highly prevalent in patients with CPP [42,44,45,46]. 

In the urological field, the main clinical entities that refer to the relationship, in some aspect of their symptomatology, with MPS are interstitial cystitis [47,48,49,50,51,52] and chronic prostatitis [45,51,52,53,54,55,56]. In both pathologies, the assessment and treatment of MTrPs is both abdominal musculature and SP musculature [45,47,48,49,50,51,52,53,54,55]. The main improvement in both cases were in pain and quality of life [45,50]. In the case of prostatitis, improvements were seen not only in those aspects, but also in urinary problems and sexual dysfunction [53,54,56].

Although fewer studies have been published in this regard, in the coloproctological sphere the relationship between PF myofascial pathology and anorectal pain is highlighted [57], and Ashrafi et al., reported the presence of MTrPs in abdominal and lumbopelvic musculature with functional constipation problems [58].

Within gynecology, dyspareunia, endometriosis, and dysmenorrhea are the disorders most frequently associated with the presence of MTrPs. Although there are no studies on the prevalence of MTrPs in each specific pathology, we do have data that estimate their prevalence rate in CPP to be between 22% and 94%, most being gynecological disorders [42].

Regarding PD in particular, Yacubovich et al., considered that the data on the prevalence of MTrPs in adolescent women with PD in the abdominal and lumbar musculature are high, triple the number of MTrPs found in women with PD compared with women without PD. For this reason, it should be taken into account in exploration and treatment [59].

In the study by Gaubeca et al., dry needling treatment was performed on women with PD on the abdominal muscles [22] and, in the study by Huang et al., invasive treatment was also performed on this muscle, although in this case it was a lidocaine infiltration [23]. In both studies, improvements in pain reduction of up to 3 points on the visual analog scale (VAS) were found, which were also maintained in the medium and long term [22,23]. A study by Langford et al., found improvements in patients with CPP after infiltration of lidocaine over MTrPs of the levator ani muscles [60]; and extant studies that compared the effects of lidocaine, botulinum toxin, and dry needling on MTrPs show similar results [61,62]. A 2013 systematic review on the use of botulinum toxin demonstrated relief in patients with CPP secondary to muscle spasm of PF muscles [63].

Although the main clinical practice guidelines indicate the need to explore the location of the pain in CPP—referring to the exploration of the PF muscles—only a recent study by Espinosa et al., provided treatment for MTrPs of this musculature through manual techniques in women with PD, also achieving clinical improvements [24]. Myofascial pain syndrome was studied by Travell and Simons [27], and other urological disorders such as prostatitis and interstitial cystitis have a high prevalence of MTrPs; their examination offers broader descriptions of referred pain [43,45,49,50,51].

There is a lack of scientifically based resources in clinical practice that can guide the development of physiotherapy treatment for MPS in women with CPP who, in many cases, in a pelviperineal evaluation, manifest referred pain from the PF muscles that they identify as their menstrual pain. Therefore, assessing the responsibility of the MTrPs in this musculature, as well as expanding the maps of referred pain that they reproduce, may offer conservative therapeutic alternatives with fewer side-effects than the main forms of treatment currently benefiting women with PD, improving their quality of life and reducing the socioeconomic and psychological impacts of this gynecological disorder. Therefore, the objective of our study was to determine whether patients suffering from PD show an alteration in local mechanosensitivity—which could be related to the presence of MTrPs in the abdominal and PF muscles—compared to women without PD. We also aimed to obtain new maps of referred pain that can elaborate on the current maps.

## 2. Material and Methods

We performed a cross-sectional descriptive study of cases and controls that assessed four groups. The evaluator was blind with respect to the condition of participants. This study meets the criteria of The Strengthening the Reporting of Observational Studies in Epidemiology (STROBE) [64], and was approved by the Research Ethics and Animal Experimentation Committee of the University of Alcalá (IEC Code: CEID/HU/2017/01).

The sample was obtained using a consecutive sampling method between October 2018 and May 2021. The study participants were recruited and assessed at the CARMASALUD Clinical and Research Center, located in Madrid, Spain.

The target population was women of childbearing age over 18 years of age. The participants had to have a regular menstrual cycle that was no longer than 35 days [65,66], and they were considered to have dysmenorrhea if they had a VAS greater than 3/10 at the time of the first assessment [22,23]. The women participated in the study voluntarily and provided written informed consent.

Women whose menstrual cycle was irregular or longer than 35 days were excluded from the study, as were women suffering from secondary dysmenorrhea (i.e., the menstrual pain was related to some gynecological pathology that caused pain, such as endometriosis or adenomyosis); those suffering from chronic pelvic pain unrelated to their menstrual cycle [22,23]; those diagnosed with fibromyalgia, since they are considered to be patients suffering from a central sensitization process where pressure pain thresholds are altered [67]; and those who had taken analgesics in the 12 h prior to the assessment, considering that, based on the pharmacokinetics of the analgesics administered, after this 12 h period, the effects of the drug no longer persist, and the drugs do not interfere with the perception of pain or compliance with questionnaires [68].

### 2.1. Procedures, Variables and Assessments

Sociodemographic and clinical data were collected through a personalized questionnaire filled out by the women themselves. They self-completed the 12-item Short Form Survey (SF-12) [69], the Mc Gill pain questionnaire [70,71,72,73], and the VAS [73].

The first evaluation was performed within the first 3 days of menstruation, in which, in addition to self-completing the questionnaires, algometry was used to record PPT values, and a manual exploration was carried out by palpation on the study musculature (i.e., the PF musculature) performed vaginally, using gloves. The muscles evaluated were: rectus abdominis, external oblique, internal oblique, quadratus lumborum, gluteus maximus, gluteus medius, gluteus medius, gluteus minimus, adductor magnus, piriformis, bulbospongiosus, ischiocavernosus, transverse perineal, puborectus, pubococcygeus, iliococcygeus, coccygeus, obturator internus, and external anal sphincter. This palpatory examination was performed to confirm the presence or absence of MTrPs. The women in the study shaded the areas of referred pain they felt during the examination on a body representation map. The second evaluation was based on the duration of the menstrual cycle of each of the women; we sought to perform the second evaluation at the time of ovulation, 14 days before the end of the menstrual cycle, given the periovulatory phase in women without contraceptive, or intermenstrual treatment in those who were under contraceptive treatment. In said intervention, the VAS was completed again, and we recorded once more the PPT, the presence of MTrPs, and the pain as referred to in the body representation map. The third and last interventions were carried out 1 week later, during which only VAS and PPT data were collected. The professional who performed the examinations was a physiotherapist with expertise in urogynecological physiotherapy and myofascial pain and with more than 10 years of experience.

The diagnosis of MTrPs was made by manual palpation following the diagnostic criteria described by Travell and Simons in 1999 [27], revised and recommended in a recent Delphi study [33]: presence of a taut band within the explored muscle; presence of a nodule or hypersensitive area to palpation located within the tense band of the explored muscle; and presence of referred pain appearing after sustained compression on the trigger point.

The indications to the participants were: “It may be that some of the points where we apply pressure cause pain; in that case, if the pain is recognized as your usual pain, you should check the box for active MTrPs, and if the pain is not recognized as usual during your menstruation, you should check the box for latent MTrPs. In some cases, in addition to the pain in the pressure area, you may notice that your pain refers to other areas; in that case, shade in the areas in the body representation map to which it spreads.”

### 2.2. Sample Size Calculation

A priori sample size calculation was carried out based on a pilot study using the same methodology, since there were no previous similar studies, by F-test for repeated measures analysis of variance (ANOVA; intra-between interaction) using G*Power 3.1.9.2 software for Windows, based on the main outcome measure of the pressure pain threshold (kg/cm^2^) in the lumbar region just below L5 from a pilot study with 4 groups (n = 20; 5 women comprised the group without dysmenorrhea and without contraceptives; 5 women comprised the group without dysmenorrhea and contraceptives; 5 women comprised the dysmenorrhea and no contraceptives group; and 5 women comprised the dysmenorrhea and contraceptives group) and 3 measurement times (proliferative phase, ovulation phase, and luteal phase), showing a partial eta^2^ of η^2^ = 0.048 corrected for Greenhouse–Geisser. Assuming an effect size of f = 0.22, an error probability α of 0.01, power (1-β error probability) of 0.90, correlation between repeated measurements of 0.5, and a correction for non-sphericity of 1, a total sample size of 84 participants (n = 21 women per group) was needed to achieve a true power of 0.91.

### 2.3. Statistical Analysis

Statistical analysis was performed using SPSS Statistics software v. 27.0 (IBM, Chicago, IL, USA; 2021).

According to the a priori sample size calculation parameters, statistical significance was set at *p* ˂ 0.01 for a 99% confidence interval (CI). For quantitative data, the Shapiro–Wilk test was used to assess normal distribution. Although some data were not normally distributed, F-tests are robust in terms of type I error and regardless of manipulated conditions, and are considered a valid option for nonparametric distributions [74]. In addition, the mean ± standard deviation (SD), with 99% CIs, was applied to describe these quantitative data. First, ANOVA was applied to compare the differences between groups of descriptive data and questionnaire results at a single measurement time. Second, a two-way ANOVA was performed for repeated measures over time (at the menstrual, ovulation, and luteal phases of the menstrual cycle) as a within-subject factor and group (group without dysmenorrhea and no contraceptives; group without dysmenorrhea and with contraceptives; dysmenorrhea and no contraception group; and dysmenorrhea and contraception group) as a between-group factor to compare VAS and PPT outcomes [74]. The significance of the Greenhouse–Geisser correction was considered when the Mauchly test rejected sphericity [75]. In addition, post hoc comparisons were made by the Bonferroni correction. The effect size of the F-tests was analyzed using the coefficients of eta squared (η^2^) and interpreted as a small effect size for the η^2^ = 0.01 coefficient, a medium effect size for η^2^ = 0.06, and a large effect size for η^2^ = 0.14 [76]. Effect size for post hoc comparisons was determined using Cohen’s *d* (*d* = *2t*⁄√*gdl*) and interpreted as very small effect size (*d* < 0.20), small effect size (*d* = 0.20–0.49), medium effect size (*d* = 0.50–0.79), and large effect size (*d* > 0.8) [75,76,77]. These data were represented by linear graphs using means with 99% CIs. A *p*-value < 0.01 was considered statistically significant for a 99% CI, and was completed with the F statistic for all ANOVA analyses and adjusted for Greenhouse–Geisser corrections [75]. Finally, the categorical data were described by frequencies and percentages (n[%]) and compared by the chi square test (χ^2^) [78]. The statistical differences were visualized as bar graphs indicating the distribution of frequencies.

## 3. Results

**Figure 1:**
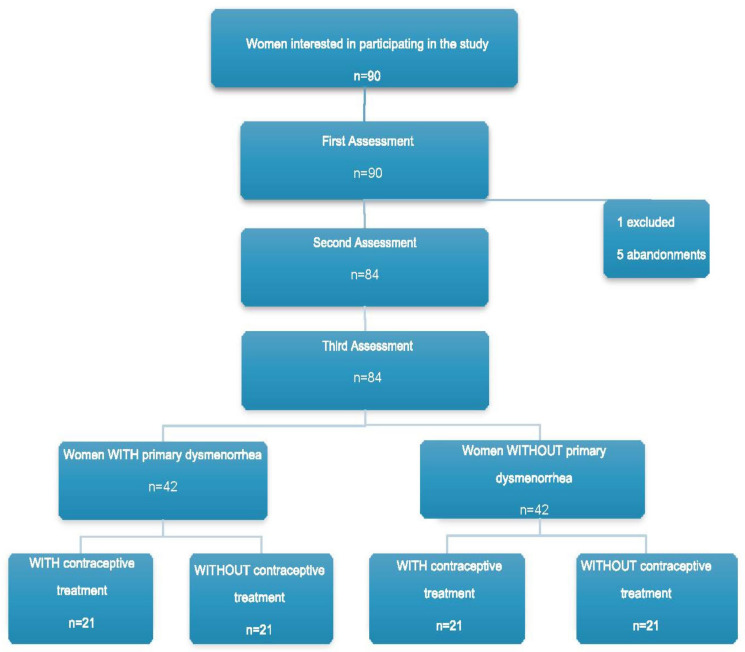
Flowchart.

A total of 90 women decided to participate in the study. Six women dropped out of the study, resulting in a final sample of 84 participants, who were divided into the following groups: women with dysmenorrhea and with contraceptive treatment (WDWC) (n = 21), women with dysmenorrhea without contraceptive treatment (WDNC) (n = 21), women without dysmenorrhea with contraceptive treatment (NDWC) (n = 21), and women without dysmenorrhea and without contraceptive treatment (NDNC) (n = 21). This process is outlined in the flowchart in Figure 1.

### 3.1. Demographics

The total sample consisted of 84 participants with a mean ± SD age of 31.20 ± 7.65 years, weight of 58.22 ± 6.50 kg, height of 1.64 ± 0.04 m, and a BMI of 21.50 ± 2.25 kg/m^2^. The one-way ANOVA did not show statistically significant differences (*p* > 0.01) between the groups, with an effect size that varied from small to medium (η^2^ = 0.041–0.124) for any of the sociodemographic variables (Table 1).

A share of 50% of the study participants were not under contraceptive treatment and, of this 50%, more than half, 29.76%, were taking oral contraceptives; 13.10% were using the vaginal ring and 7.14% had an IUD implanted. Half of the sample reported back pain on some occasion, and 28.57% reported pelvic pain at times unrelated with menstruation. Statistically significant data (*p* < 0.01) were found for gastrointestinal disturbances such as diarrhea, constipation, nausea, vomiting, or discomfort, which appeared in 63.10% of participants during menstruation, with only 15.48% suffering from them outside the menstrual bleeding period. Dyspareunia was found in 29.76% of women and 69.05% considered they had menstrual pain. Statistically significant data (*p* < 0.001) were found for menstrual treatment, with 35.71% of participants not needing treatment to relieve menstrual symptoms. The most widely used treatment to alleviate these symptoms was medication, which was used in 30.95% of cases, followed by the combination of heat and medication, used by 28.57% of participants. The remaining variables from the qualitative clinical descriptive analysis did not show statistically significant differences (*p* > 0.001). These data are shown in Table 2.

### 3.2. Quality of Life

One-way ANOVA revealed statistically significant differences (*p* < 0.01) between the groups, with a large effect size (η^2^ = 0.168–0.180) for the Physical Health domain and a high total SF-12 score (Table 3). However, no significant differences were found (*p* > 0.01) between the groups, with a medium effect size (η^2^ = 0.104–0.106) for the Mental Health domain of the SF-12 questionnaire.

Post hoc comparisons between groups for the Physical Health domain of the SF-12 questionnaire showed statistically significant differences (*p* = 0.001), with a large effect size (*d* = 1.191–1.206) for the NDWC vs. WDNC comparison, showing a higher percentage for the Physical Health domain in the NDWC group compared with the WDNC group. Similarly, these post hoc comparisons for the total score of the SF-12 questionnaire showed statistically significant differences (*p* < 0.01), with a large effect size (*d* = 1.260–1.274) for the NDWC vs. WDNC comparison, showing a higher score the total SF-12 score in the NDWC group relative to the WDNC group. The rest of the post hoc comparisons showed no significant differences.

### 3.3. Pain

#### 3.3.1. The McGill Pain Questionnaire

Statistically significant differences (*p* < 0.01) were found between the groups, with a large effect size (η^2^ = 0.262–0.459) for all domains of the McGill Pain Questionnaire (PRI-S, PRI-A, PRI-E, PRI-Total, Number of words, and PPI), as shown in Table 4.

The post hoc comparisons showed a significantly higher score in the NDNC group for the PRI-E (*p* < 0.01), with a large effect size (*d* = 1.090). The WDNC group had a significantly higher score than other groups in all domains (PRI-S, PRI-A, PRI-E, PRI-Total, Number of words, and PPI; *p* < 0.01), with a large effect size (*d* = 0.953–1.652). Similarly, post hoc comparisons between the NDWC and WDNC groups and between the NDWC and WDWC groups showed significant differences (*p* < 0.01) in all domains, with a higher score for the WDNC and WDWC groups, with a large effect size. (*d* = 1.442–2.410 and *d* = 1.269–2.163, respectively). The WDWC group had a significantly higher score than the NDCA group for the domains PRI-S, PRI-Total, and Number of words, with a large effect size (*d* = 1.033–1.055). The WDNC had a significantly higher score than the WDWC group in the PPI domain only (*p* < 0.01), with a large effect size (*d* = 0.974).

#### 3.3.2. Visual Analog Scale

**Figure 2:**
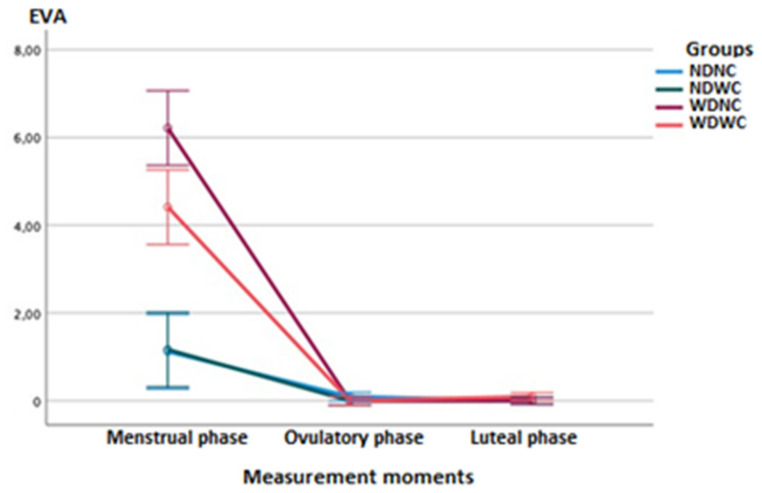
Linear graphs of the means completed with the error bars at 99% of the Cl, for the pain intensity variable (VAS scale) that show the differences between the groups (NDNC, NDWC, WDNC, WDWC) and the measurement moments (menstrual phase, ovulatory phase, and luteal phase).

Regarding between-group comparisons, the two-way ANOVA test (3 measurement moments × 4 groups) of repeated measures showed statistically significant differences (*p* < 0.001; *F* = 58.519; η^2^ = 0.687) between the groups, with a large effect size for pain intensity measurements using the VAS (Figure 2). Post hoc comparisons of VAS score between groups in the menstrual phase showed statistically significant differences (*p* ≤ 0.001) with a large effect size (*d* = 2.052–3.776), indicating a lower intensity of pain in the NDNC group with respect to the WDNC group, in the NDNC group with respect to the WDWC group, and in the NDWC group with respect to the WDNC group; however, a greater pain intensity in the NDWC group vs. the WDWC group was found. The remaining post hoc comparisons, in the ovulatory phase and the luteal phase, indicated no further significant differences.

Regarding intra-group comparisons, the ANOVA test showed statistically significant differences (*p* < 0.001; *F* = 377.092; η^2^ = 0.827) between the different measurement moments, with a large effect size for pain intensity measurements using the VAS. Post hoc comparisons for pain intensity of the NDNC and NDWC groups showed statistically significant differences (*p* < 0.01), with a large effect size (*d* = 1.367–1.763), indicating greater pain intensity in the menstrual phase relative to the ovulatory and luteal phases. Post hoc comparisons in the WDNC and WDWC groups showed statistically significant differences (*p* < 0.001), with a large effect size (*d* = 3.299–5.196), also reflecting a greater intensity of pain in the menstrual vs ovulatory and luteal phases.

### 3.4. Mechanosensitivity

#### 3.4.1. Between-Group Comparisons

**Figure 3:**
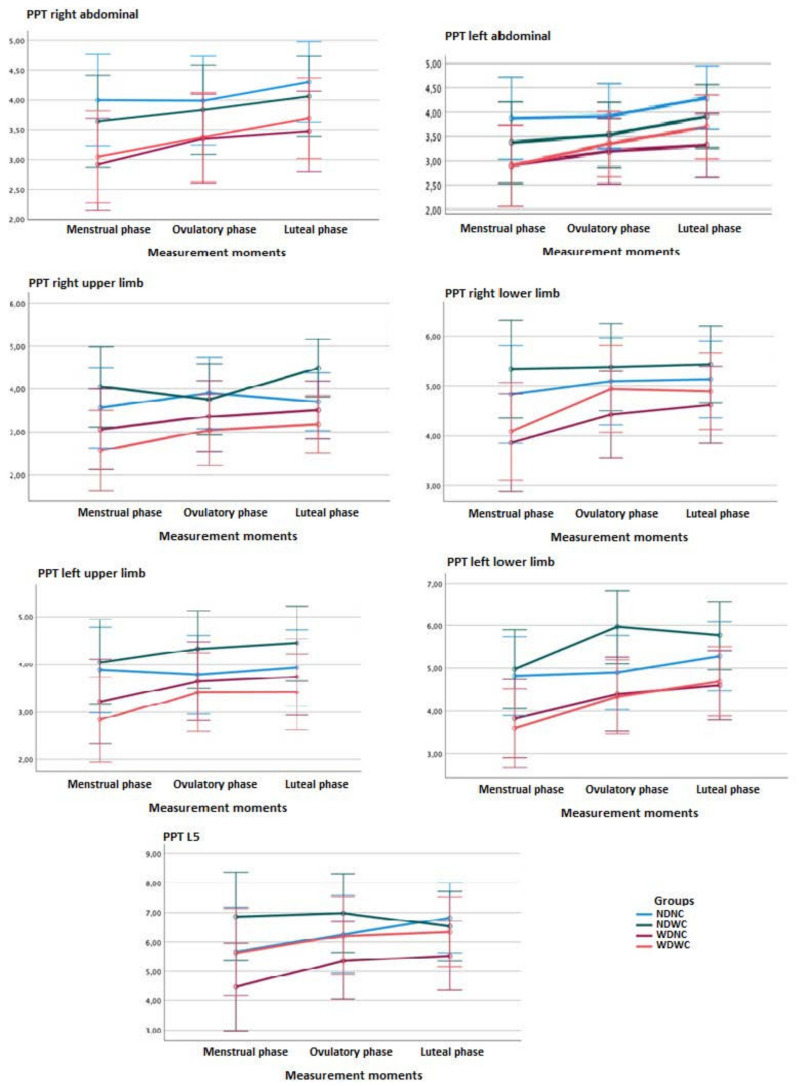
Linear graphs of the means completed with the error bars at 99% of the Cl, for the variable pressure pain threshold (PPT) that show the differences in the different measurement zones between the groups and the measurement moments (menstrual phase, ovulatory phase and luteal phase).

Regarding between-group comparisons, the two-way ANOVA test (3 measurement moments × 4 groups) of repeated measures did not show statistically significant differences (*p* > 0.01; *F* = 1.160; η^2^ = 0.019) between the groups, with a small effect size for PPT measurements in the abdominal region and L5 (Figure 3). Post hoc comparisons of right abdominal PPT between groups at different measurement phases (menstruation, ovulation, and luteal) revealed no statistically significant differences.

For measurements in areas furthest from the upper and lower extremity pain, repeated measures two-way ANOVA (3 time-points × 4 groups) showed no statistically significant difference (*p* > 0.01, *F* = 1.389; η^2^ = 0.032) between groups, with a small effect size, as shown in Figure 3. Post hoc comparisons of the PPT in the right upper limb between the NDWC and WDWC groups in the luteal phase showed significant differences (*p* < 0.01) with a large effect size (*d* = 1.133), and post hoc comparisons of PPT in the left lower limb between groups in the ovulatory phase showed significant differences (*p* < 0.01), with a large effect size (*d* = 0.944–1.049), indicating a lower intensity of pain in the NDWC vs. WDNC group and in the NDWC vs. WDWC group.

#### 3.4.2. Within-Group Comparisons

Regarding the intra-group comparisons, the ANOVA test revealed significant differences (*p* < 0.001; *F* = 14.381; η^2^ = 0.152) between the different measurement moments, with a small effect size for the PPT measurements in the right abdominal region and L5. Significant differences (*p* < 0.001; *F* = 11.627; η^2^ = 0.127) were also found between the different measurement moments, with a small effect size for the PPT measurements in the left abdominal region (Figure 3).

Post hoc comparisons for the PPT in the right abdominal area of the NDNC, NDWC, and WDNC groups indicated no significant differences between the measurement time-points. Significant differences (*p* < 0.01) were found for the PPT in the right abdominal region in the WDWC group, with a medium effect size (*d* = 0.556), indicating a greater intensity of the PPT in the menstrual vs. the luteal phase, but not the ovulatory phase. The rest of the post hoc comparisons for all groups in the left abdominal region and L5 did not reveal any further significant differences (Figure 3).

For the measurements in regions farthest from the pain in the upper and lower extremities, no significant differences were found (*p* > 0.01; *F* = 5.996; η^2^ = 0.054) between the different measurement time-points, with a small effect size for upper limb PDU measurements. However, significant differences were found (*p* = 0.001, *F* = 8.313, η^2^ = 0.094) between the measurement time-points, with a medium effect size for right lower-limb PDU measurements, and those in the left lower limb (*p* < 0.001; *F* = 18.175; η^2^ = 0.185), with a large effect size.

Post hoc comparisons indicated significant differences for PPT in the extremities. In the right upper limb, the significant data were in the NDWC group (*p* < 0.01), with a medium effect size (*d* = 0.574), indicating a higher PPT in the ovulatory vs. luteal phase. Post hoc comparisons for these PPT of the NDNC, WDNC, and WDWC groups showed no significant differences between the measurement time-points for the right upper limb. For the PPT of the right lower limb of the WDNC group, a significantly lower PPT was found (*p* = 0.01) in the menstrual vs. luteal phase, with a small effect size (*d* = 0.474). Similarly, a significantly lower PPT in the WDWC group was found (*p* < 0.01) in the menstrual vs. luteal phase, with a medium effect size (*d* = 0.593). In the lower left limb of the NDWC group, a significantly lower PPT in the NDWC group was found (*p* < 0.01) in the menstrual vs. ovulatory phase, with a medium effect size (*d* = 0.559). Similarly, a significantly lower PPT in the WDWC group was found (*p* < 0.01) in the menstrual vs. luteal phase, with a large effect size (*d* = 0.953) (Figure 3).

### 3.5. Presence of MTrPs

The prevalence of MTrPs in the muscles during the menstrual phase is shown in Table 5 and Table 6. A significantly higher prevalence of MTrPs in the rectus abdominis muscle was found (*p* < 0.01) in the WDNC and WDWC groups (52.38% and 61.90%, respectively). A significantly higher prevalence of active MTrPs in the gluteus maximus was found in the WDWC group (23.80%). A significantly higher prevalence of active MTrPs in the ischiocavernosus muscle was found in the WDNC and WDWC groups, whereas MTrPs were absent in the NDNC and NDWC groups. Furthermore, a significantly higher prevalence of active MTrPs in the pubococcygeus muscle was found in the WDNC and WDWC groups (66.66%). The remaining muscles studied showed no significant differences, although the iliococcygeus muscle presented active MTrPs in more than half of the total sample (69.04%).

The prevalence of MTrPs in the muscles during the periovulatory or intermenstrual phases is shown in Table 7 and Table 8. A significantly higher prevalence of MTrPs was found in the WDNC and WDWC groups for the ischiocavernosus muscle (66.66% and 71.4%, respectively). A high prevalence of active MTrPs in the iliococcygeus muscle was also found in the WDNC and WDWC groups (85.71%); MTrPs were present in 60.71% of the total sample. Furthermore, there is a greater presence of latent MTrPs in the external anal sphincter in the WDWC group.

### 3.6. Referred Pain Areas

#### Rectus Abdominis Muscle

**Figure 4:**
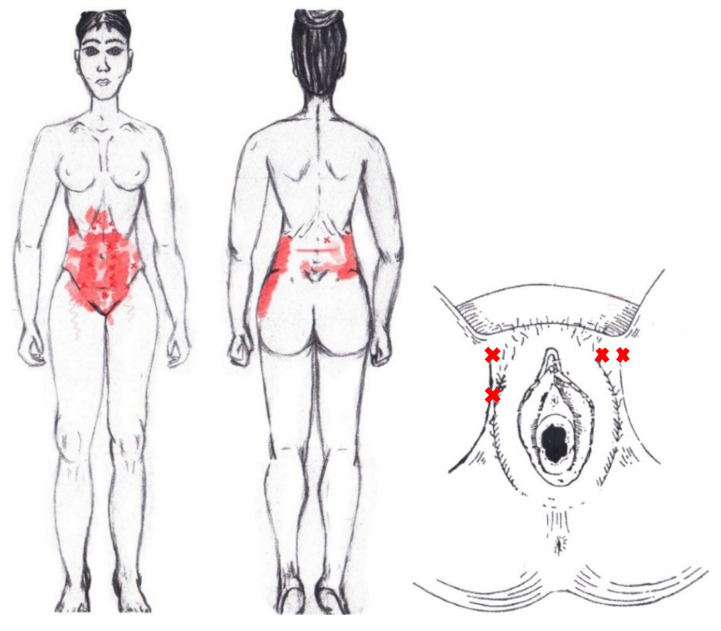
Graphical simulation of referred pain areas of the rectus abdominis muscle.

Statistically significant results were found during the menstrual phase for the areas of referred pain in the flank (64.29%), iliac fossa (40.48%), pubis (11.90%), and groin (8.33%), with a higher frequency of these areas of referred pain in the WDNC group. In the rest of the referred pain areas, and in all referred pain areas during the ovulatory phase, no significant results were found for the rectus abdominis muscle. Figure 4 shows a graphical simulation of the representation of the referred pain, showing with greater intensity of color the zones where it was referred with greater frequency and, with less intensity, those with less frequency.

The frequency at the areas of referred rectus abdominis muscle pain in the menstrual phase and in the ovulatory phase is shown in Table 9 and Table 10, respectively.

### 3.7. External Oblique Muscle

**Figure 5:**
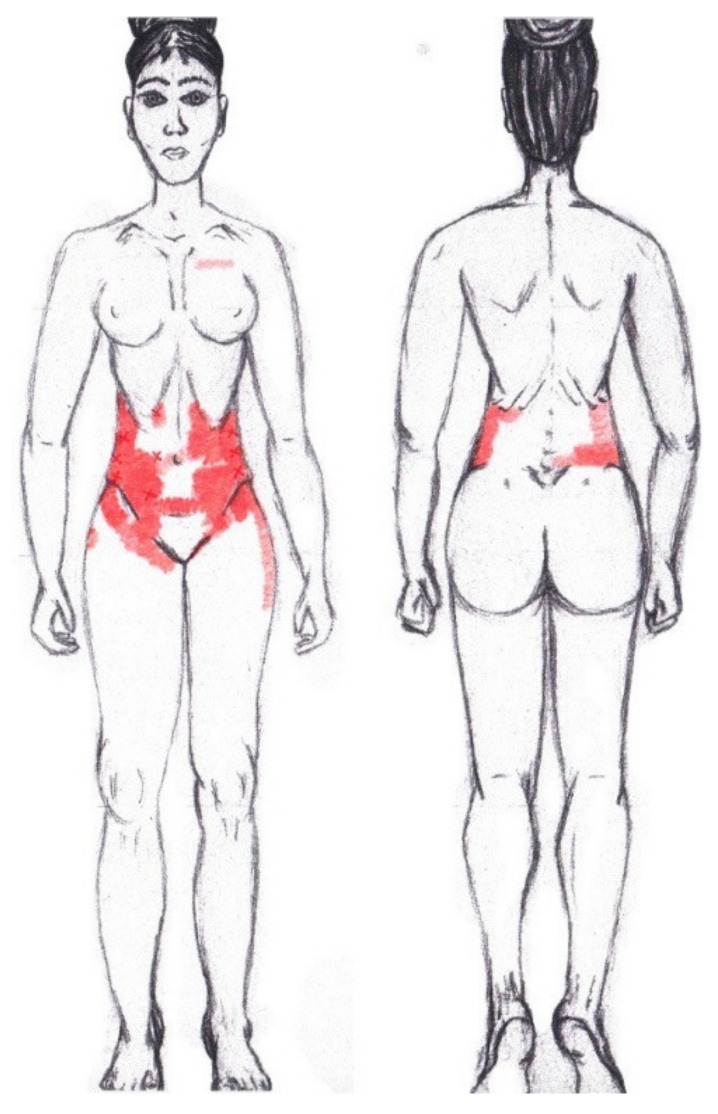
Graphical simulation of referred pain areas of the external oblique muscle.

No significant results were found during either the menstrual or ovulatory phase in any of the areas of referred pain for the external oblique muscle. Figure 5 shows a graphical simulation of the representation of the referred pain zones, showing with greater intensity of color the zones where it was referred with greater frequency and, with less intensity, those with less frequency.

The frequency at the areas of referred external oblique muscle pain in the menstrual phase and in the ovulatory phase is shown in Table 9 and Table 10, respectively.

### 3.8. Internal Oblique Muscle

**Figure 6:**
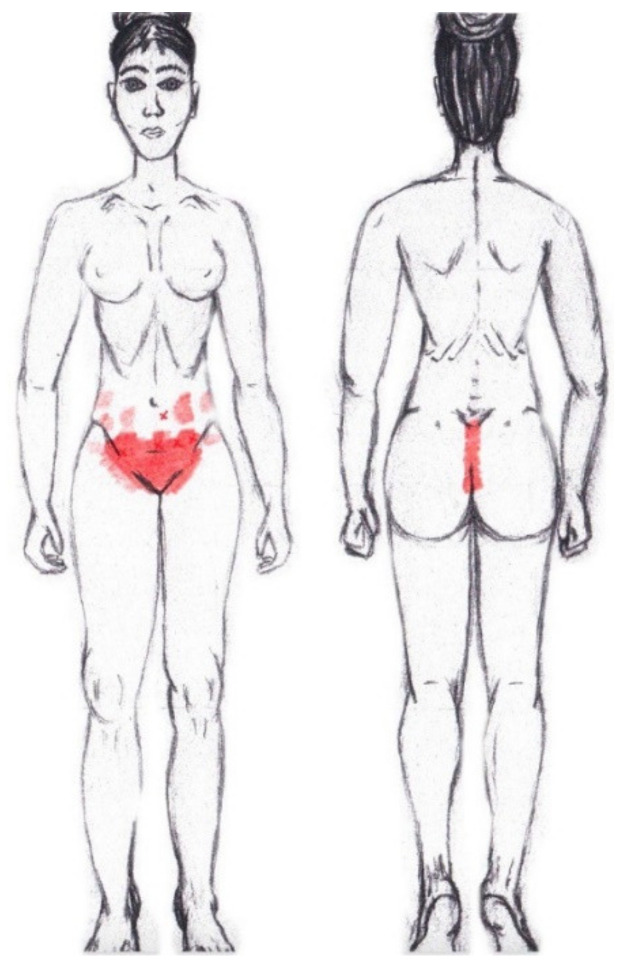
Graphical simulation of referred pain areas of the internal oblique muscle.

A significantly higher frequency of the referred pain areas of the pubis (11.90%) and groin (16.67%) were found in the WDNC group. In the rest of the referred pain areas and in all referred pain areas during the ovulatory phase, no significant results were found for the internal oblique muscle. Figure 6 shows a graphical simulation of the representation of the referred pain zones, showing with greater intensity of color the zones where it was referred with greater frequency and, with less intensity, those with less frequency.

The frequency at the areas of referred internal oblique muscle pain in the menstrual phase and in the ovulatory phase is shown in Table 9 and Table 10, respectively.

### 3.9. Adductor Magnus Muscle

**Figure 7:**
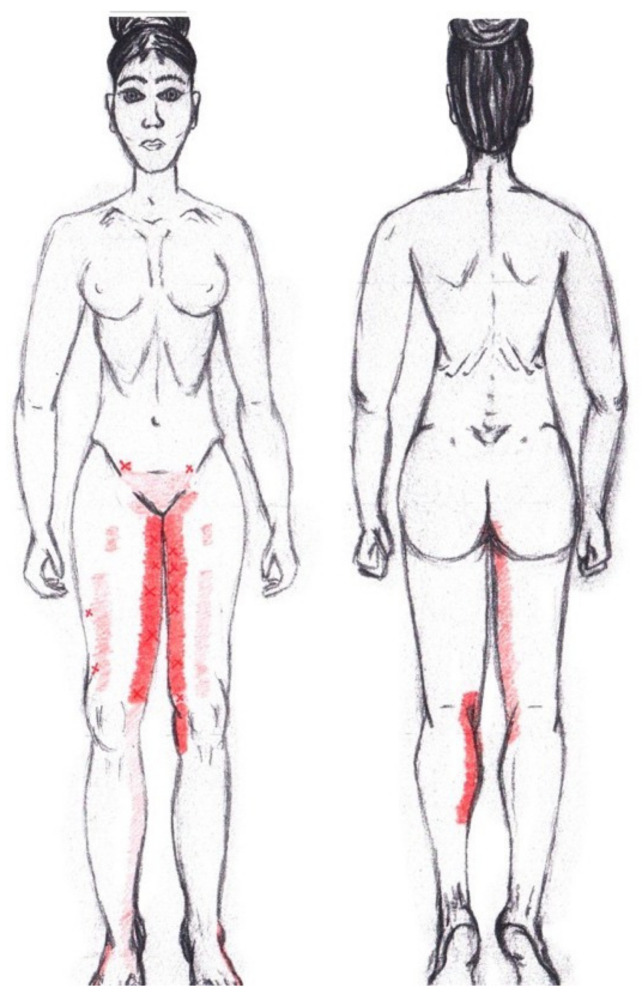
Graphical simulation of referred pain areas of the adductor magnus muscle.

No significant results were found in either the menstrual or ovulatory phase in any of the areas of referred pain for the adductor magnus muscle. Figure 7 shows a graphical simulation of the representation of the referred pain zones, showing with greater intensity of color the zones where it was referred more frequently and, with less intensity, those with less frequency.

The frequency at the areas of referred adductor magnus muscle pain in the menstrual phase and in the ovulatory phase is shown in Table 9 and Table 10, respectively.

### 3.10. Gluteus Maximus Muscle

**Figure 8:**
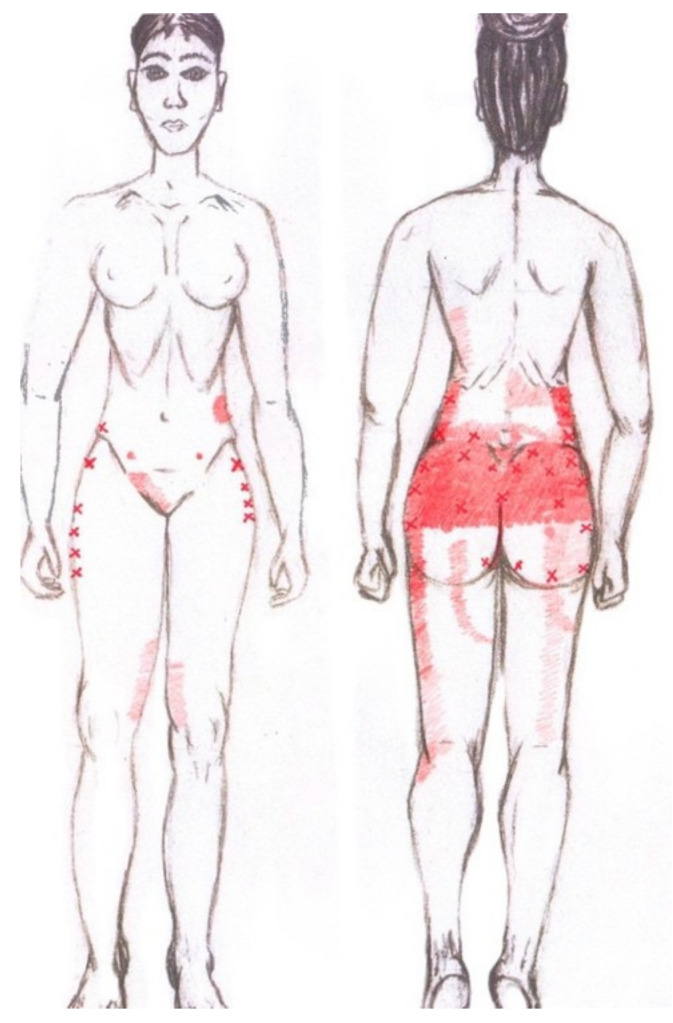
Graphical simulation of referred pain areas of the gluteus maximus muscle.

No significant results were found in either the menstrual or ovulatory phase in any of the areas of referred pain for the gluteus maximus muscle. Figure 8 shows a graphical simulation of the representation of the referred pain zones, showing with greater intensity of color the zones where it was referred with greater frequency and, with less intensity, those with less frequency.

The frequency at the areas of referred gluteus maximus muscle pain in the menstrual phase and in the ovulatory phase is shown in Table 9 and Table 10, respectively.

### 3.11. Gluteus Medius Muscle

**Figure 9:**
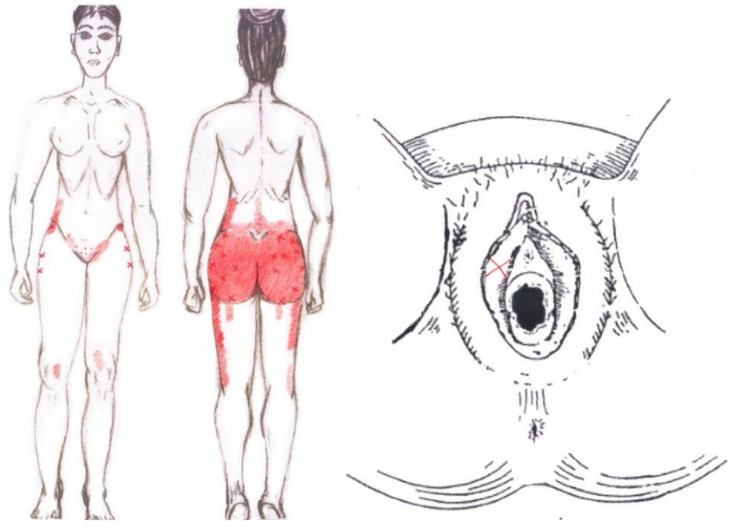
Graphical simulation of referred pain areas of the gluteus medius muscle.

No significant data were found in either the menstrual or ovulatory phase in any of the areas of referred pain for the gluteus medius muscle. Figure 9 shows a graphical simulation of the representation of the referred pain zones, showing with greater intensity of color the zones where it was referred with greater frequency and, with less intensity, those with less frequency.

The frequency at the areas of referred gluteus medius muscle pain in the menstrual phase and in the ovulatory phase is shown in Table 9 and Table 10, respectively.

### 3.12. Gluteus Minimus Muscle

**Figure 10:**
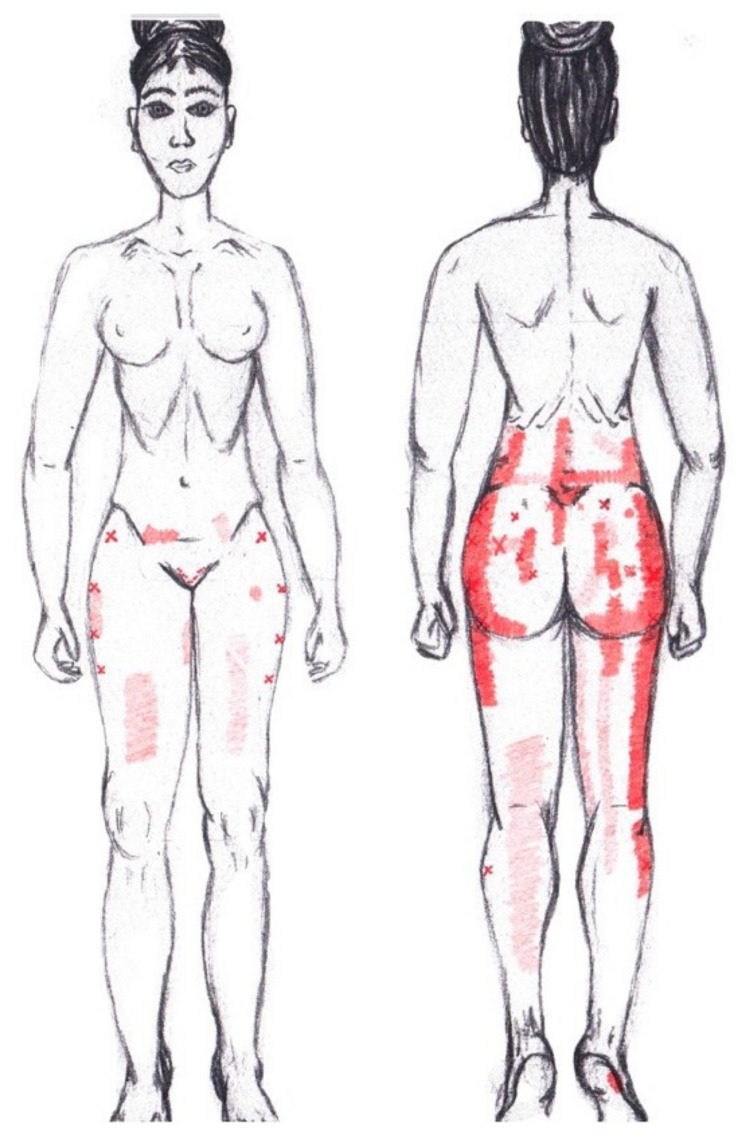
Graphical simulation of referred pain areas of the gluteus minimus muscle.

No statistically significant results emerged from either the menstrual or ovulatory phase in any of the areas of referred pain for the gluteus minimus muscle. Figure 10 shows a graphical simulation of the representation of the referred pain zones, showing with greater intensity of color the zones where it was referred more frequently and, with less intensity, those with less frequency.

The frequency at the areas of referred gluteus minimus muscle pain in the menstrual phase and in the ovulatory phase is shown in Table 9 and Table 10, respectively.

### 3.13. Quadratus Lumborum Muscle

**Figure 11:**
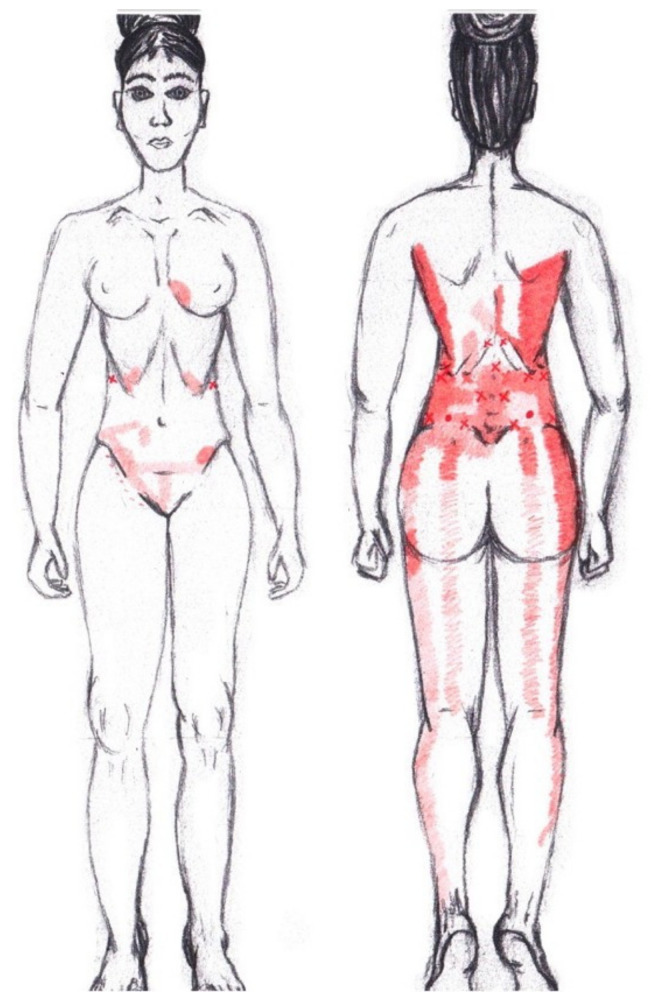
Graphical simulation of referred pain areas of the quadratus lumborum muscle.

No significant results were found in either the menstrual or ovulatory phase in any of the areas of referred pain for the quadratus lumborum muscle. Figure 11 shows a graphical simulation of the representation of the referred pain zones, showing with greater intensity of color the zones where it was referred more frequently and, with less intensity, those with less frequency.

The frequency at the areas of referred quadratus lumborum muscle pain in the menstrual phase and in the ovulatory phase is shown in Table 9 and Table 10, respectively.

### 3.14. Ischiocavernosus Muscle

**Figure 12:**
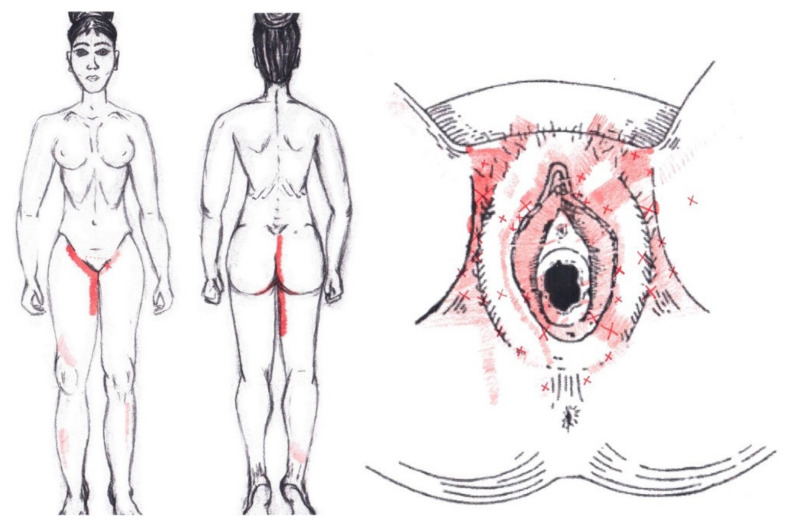
Graphical simulation of referred pain areas of the ischiocavernosus muscle.

No significant findings were found in either the menstrual or ovulatory phase in any of the areas of referred pain for the ischiocavernosus muscle. Figure 12 shows a graphical simulation of the representation of the referred pain zones, showing with greater intensity of color the zones where it was referred with greater frequency and, with less intensity, those with less frequency.

The frequency at the areas of referred ischiocavernosus muscle pain in the menstrual phase and in the ovulatory phase is shown in Table 11 and Table 12, respectively.

### 3.15. Bulbospongiosus Muscle

**Figure 13:**
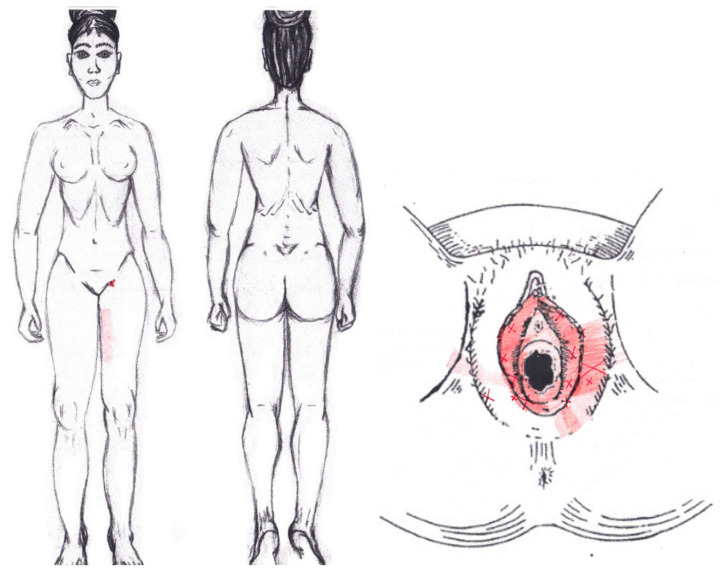
Graphical simulation of referred pain areas of the bulbospongiosus muscle.

No significant results were found in either the menstrual or ovulatory phase in any of the areas of referred pain for the bulbospongiosus muscle. Figure 13 shows a graphical simulation of the representation of the referred pain zones, showing with greater intensity of color the zones where it was referred with greater frequency and, with less intensity, those with less frequency.

The frequency at the areas of referred bulbospongiosus muscle pain in the menstrual phase and in the ovulatory phase is shown in Table 11 and Table 12, respectively.

### 3.16. Transverse Perineal Muscle

**Figure 14:**
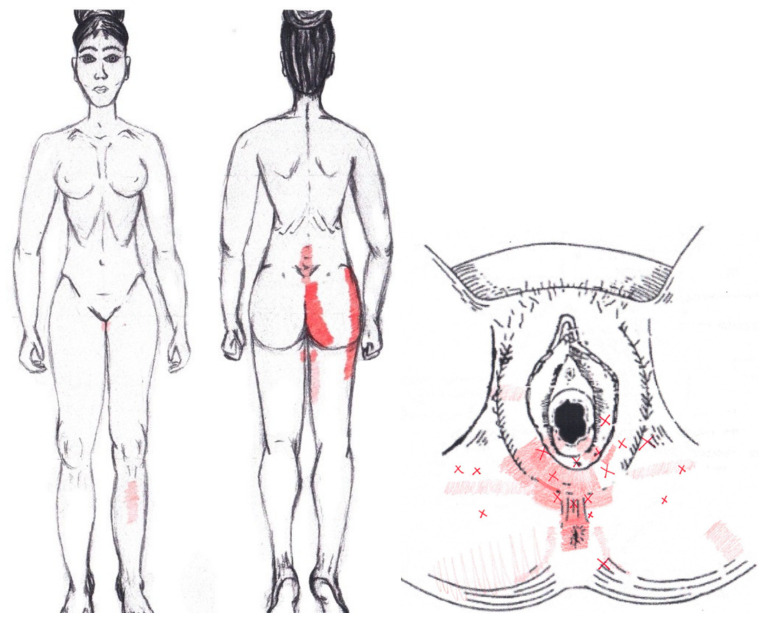
Graphical simulation of referred pain areas of the transverse perineal muscle.

No significant results were found in either the menstrual or ovulatory phase in any of the areas of referred pain for the transverse perineal muscle. Figure 14 shows a graphical simulation of the representation of the referred pain zones, showing with greater intensity of color the zones where it was referred more frequently and, with less intensity, those with less frequency.

The frequency at the areas of referred transverse perineal muscle pain in the menstrual phase and in the ovulatory phase is shown in Table 11 and Table 12, respectively.

### 3.17. Puborectalis Muscle

**Figure 15:**
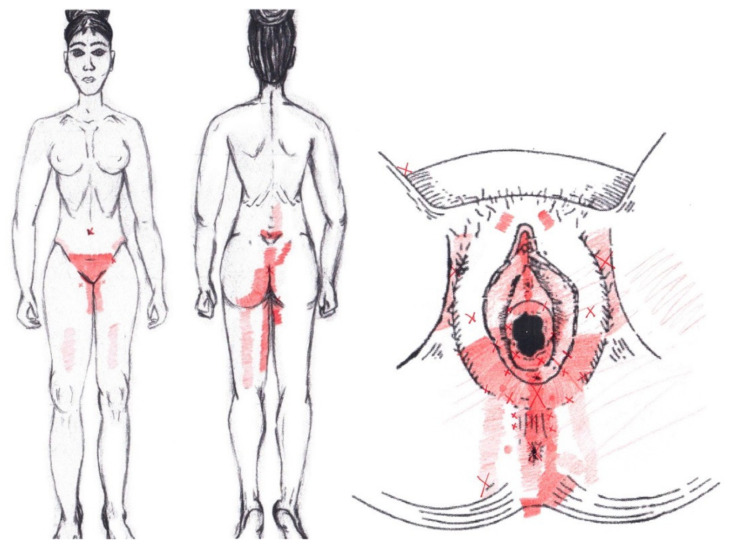
Graphical simulation of referred pain areas of the puborectalis muscle.

Statistically significant data were found in the ovulatory phase for the areas of referred groin pain, present in the rectum only in 7.14% of the women and felt almost exclusively in the NDNA group. It was identified by 4.76% of women in its entirety in the SDSA group, and it was reported in the anus by 23.81% of respondents, most from the SDSA group. In the rest of the referred pain areas in the ovulatory phase, and in all of referred pain areas in the menstrual phase, no significant results were found for the puborectalis muscle (Figure 15).

The frequency at the areas of referred puborectalis muscle pain in the menstrual phase and in the ovulatory phase is shown in Table 11 and Table 12, respectively.

### 3.18. Pubococcygeus Muscle

**Figure 16:**
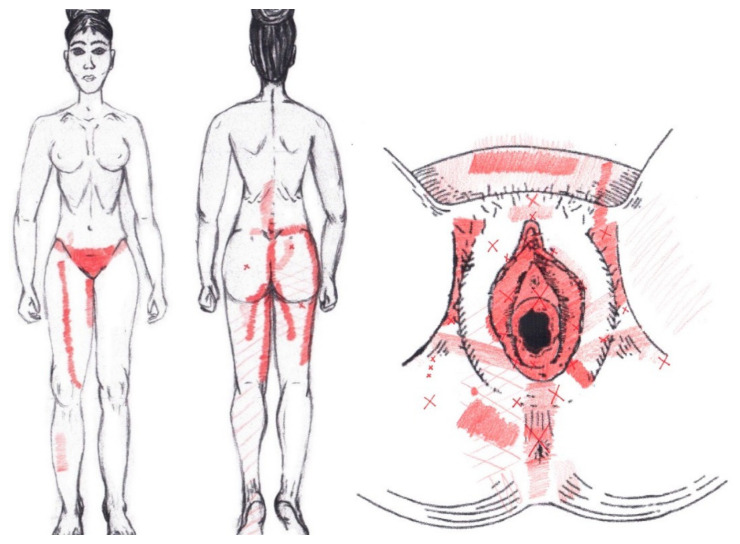
Graphical simulation of referred pain areas of the pubococcygeus muscle.

No statistically significant results were found in either the menstrual or ovulatory phase in any of the areas of referred pain for the pubococcygeus muscle. Figure 16 shows a graphical simulation of the representation of the referred pain zones, showing with greater intensity of color the zones where it was referred with greater frequency and, with less intensity, those with less frequency.

The frequency at the areas of referred pubococcygeus muscle pain in the menstrual phase and in the ovulatory phase is shown in Table 11 and Table 12, respectively.

### 3.19. Iliococcygeus Muscle

**Figure 17:**
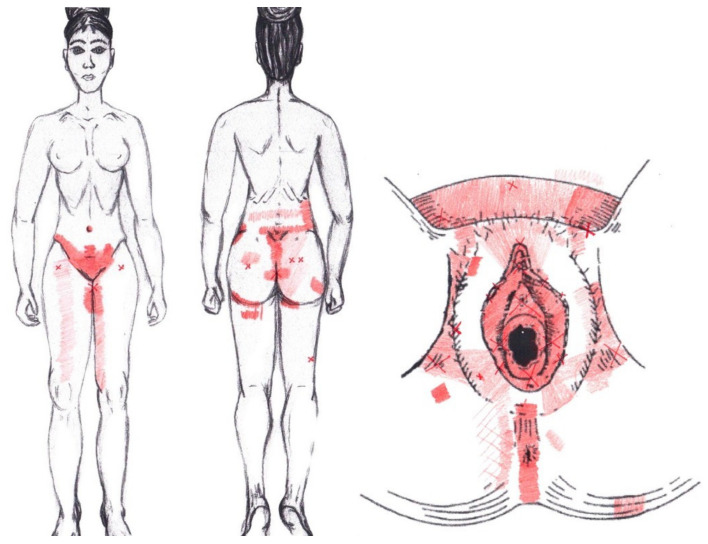
Graphical simulation of referred pain areas of the iliococcygeus muscle.

Statistically significant results were found in the ovulatory phase for the area of referred pain from the hypogastrium, which was present in 50% of respondents, most from the SDNA and SDSA groups. In the other referred pain areas in the ovulatory phase, and in all referred pain areas in the menstrual phase, no significant results were found for the iliococcygeus muscle (Figure 17).

The frequency at the areas of referred iliococcygeus muscle pain in the menstrual phase and in the ovulatory phase is shown in Table 11 and Table 12, respectively.

### 3.20. Coccygeus Muscle

**Figure 18:**
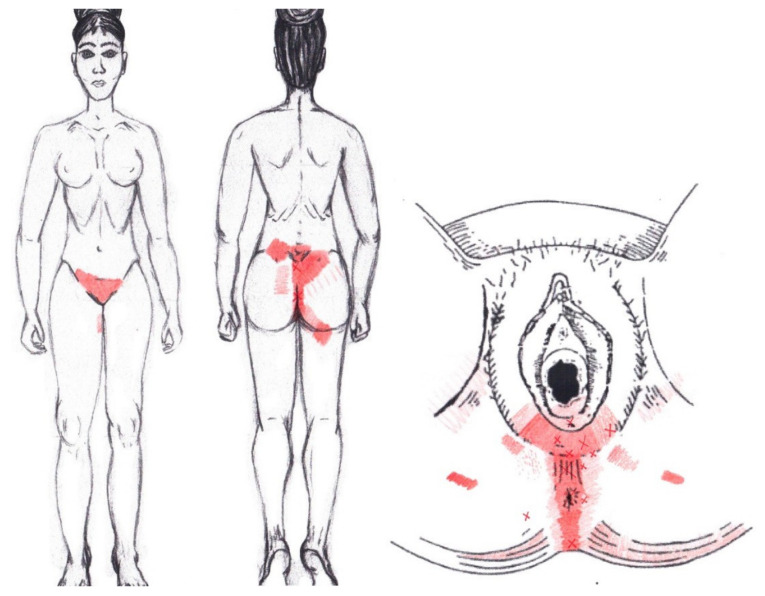
Graphical simulation of referred pain areas of the coccygeus muscle.

No significant data were found in either the menstrual or ovulatory phase in any of the areas of referred pain for the coccygeus muscle. Figure 18 shows a graphical simulation of the representation of the referred pain zones, showing with greater intensity of color the zones where it was referred with greater frequency and, with less intensity, those with less frequency.

The frequency at the areas of referred coccygeus muscle pain in the menstrual phase and in the ovulatory phase is shown in Table 11 and Table 12, respectively.

### 3.21. External Anal Sphincter Muscle

**Figure 19:**
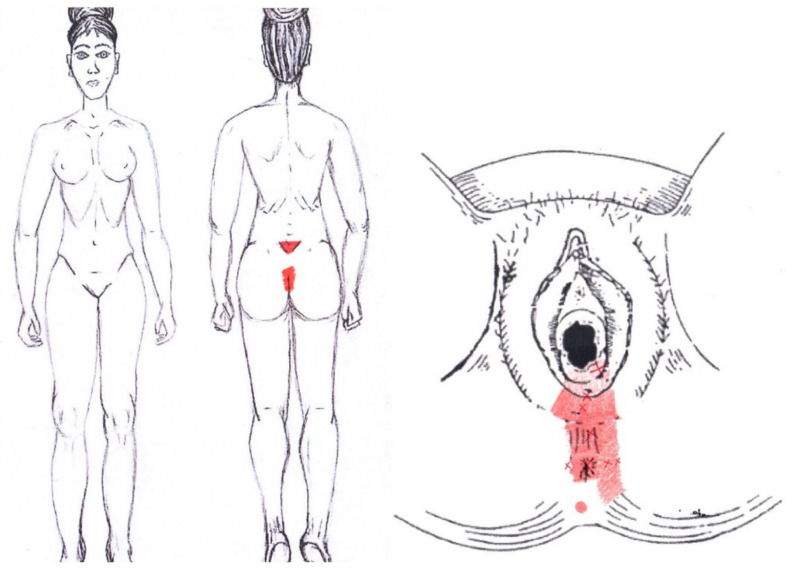
Graphical simulation of referred pain areas of the external anal sphincter muscle.

No significant data were found in either the menstrual or ovulatory phase in any of the areas of referred pain for the external anal sphincter muscle. Figure 19 shows a graphical simulation of the representation of the referred pain zones, showing with greater intensity of color the zones where it was referred more frequently and, with less intensity, those with less frequency.

The frequency at the areas of referred external anal sphincter muscle pain in the menstrual phase and in the ovulatory phase is shown in Table 11 and Table 12, respectively.

### 3.22. Obturator Internus Muscle

**Figure 20:**
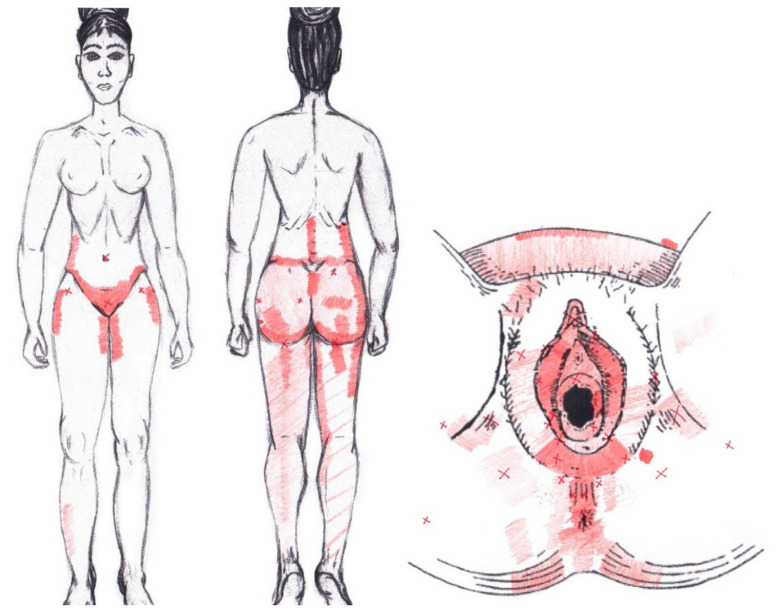
Graphical simulation of referred pain areas of the obturator internus muscle.

Statistically significant data were found in the ovulatory phase for the area of referred pain from the vulva, being present in 39.29% of the women, and its proportion being higher in the SDNA group. In the rest of the referred pain areas in the ovulatory phase, and in all referred pain areas in the menstrual phase, no statistically significant results were found for the obturator internus muscle (Figure 20).

The frequency at the areas of referred obturator internus muscle pain in the menstrual phase and in the ovulatory phase is shown in Table 11 and Table 12, respectively.

### 3.23. Piriformis Muscle

**Figure 21:**
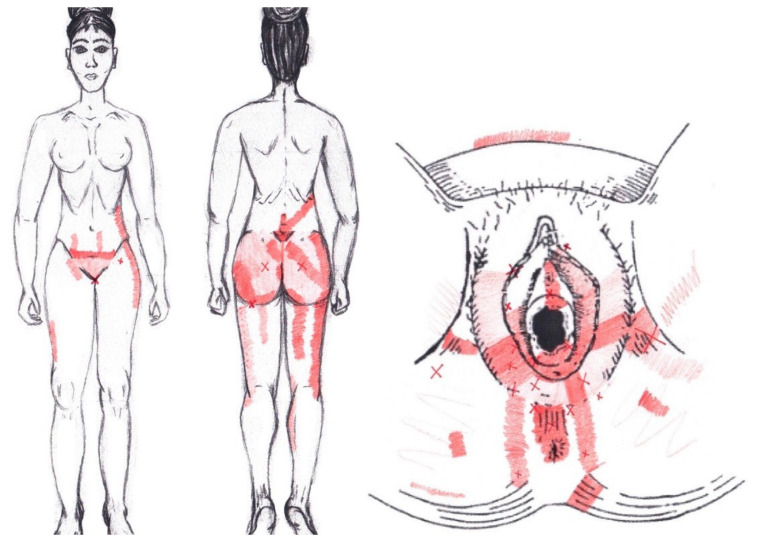
Graphical simulation of referred pain areas of the piriformis muscle.

No significant data were found in either the menstrual or ovulatory phase in any of the areas of referred pain for the piriformis muscle. Figure 21 shows a graphical simulation of the representation of the referred pain zones, showing with greater intensity of color the zones where it was referred more frequently and, with less intensity, those with less frequency.

The frequency at the areas of referred piriformis muscle pain in the menstrual phase and in the ovulatory phase is shown in Table 9 and Table 10, respectively.

## 4. Discussion

Our study sought to provide information on the possible relationship between PD and the presence of MPS, considering not only the existence of both active and latent MTrPs, but also variables such as altered mechanosensitivity to pressure and its relationship with the central sensitization process, and the description of referred pain maps of the study muscles; these factors are usually associated with the presence of MTrPs. The data were obtained from the comparison of women with and without this gynecological disorder and who were or were not receiving contraceptive treatment.

In addition to the clear difference in VAS scores between the groups with and without pain during the menstrual phase, our results indicate that the affective, emotional, and sensory descriptors collected by the McGill Pain Questionnaire yielded higher scores in groups with PD, when they are not receiving contraceptive treatment; lower scores were found in women without PD who were receiving contraceptive treatment. By comparison, there seems to be an impairment in the quality of life in women with PD who were not receiving contraceptive treatment, mainly with respect to physical health.

Despite these differences in pain and quality of life during menstruation in women with PD, these differences were not as clearly reflected in the mechanosensitivity data presented by women in the menstrual phase compared with those with and without menstrual pain. In the mechanosensitivity data, we only found differences in some of the measurement time-points collected between the groups receiving contraceptive treatment. Where we did find greater differences was in the intragroup analysis, which revealed differences between the menstrual phase and the luteal phase. The significant differences that appeared in the PPT in the periovulatory or intermenstrual phase occurred only in the group of women without PD and who were under contraceptive treatment.

The prevalence of MTrPs during menstruation exceeded 80% of the total sample for the deep muscles of the PF, and for muscles such as the rectus abdominis, external oblique, quadratus lumborum, and piriformis; elevated prevalence rates during the periovulatory or intermenstrual phase were found only in the case of the rectus abdominis muscle, with a high prevalence rate of latent MTrPs; and in the case of the iliococcygeus muscle, with a high prevalence rate of active MTrPs, predominantly among the participants with PD.

A record of referred pain areas was made to find a body representation associated with the presence of MTrPs in the different study muscles. The referred pain zones obtained in this study are considerably broader than the patterns previously described for the PF musculature.

### 4.1. Sociodemographic Factors and Clinical Data

In our study, no significant effects were found in terms of the sociodemographic variables collected, such as age or BMI, nor in the data on the menstrual cycle, such as the length of the cycle, duration of menstruation, and the onset of menstruation (menarche). The available literature continues to investigate the possible risk factors associated with dysmenorrhea, since a lack of consensus remains on some aspects. Although no differences in age groups were observed in our study, the vast majority of extant studies focused on young populations—overwhelmingly university students or those under 25 years of age—with very limited information on older women [19,79]. Data about weight and BMI are also mixed, although several studies focused on the greater predisposition in women with low weight or who have lost or attempted weight loss [19,80]. Latthe et al., found that women with a BMI < 20 kg/m^2^ report greater pain intensity [81], which, according to Nalan et al., who obtained similar results, could be due to the fact that low body fat would affect the normal ovulation and the menstrual cycle, causing an excessive release of PGs, which are considered the source of menstrual pain [80].

Regarding the characteristics of the menstrual cycle, other studies relate a greater severity of menstrual pain with longer periods, irregular cycles, or menarche onset before 12 years of age [9,80,81,82]. In the rest of the clinical variables, we found no significant results in our study, except in the gastrointestinal alterations that women present during the days of menstruation and that are absent outside of menstruation, such as nausea, discomfort, and—mainly—changes in the frequency of passing stool. The data on gastrointestinal disorders, such as constipation and diarrhea, are consistent in most studies, not only on PD, but also on premenstrual syndrome and menstruation in general [20,83,84].

From the data collected on the number of pregnancies and deliveries, and on their subsequent relationship with pain, no significant results were found between the groups. However, it should be noted that only a small percentage of the sample (19%) was under this condition. The most notable was an improvement in menstrual symptoms after childbirth, a condition more commonly described in other investigations [9]. Regarding physical activity and its effects on menstrual symptoms, no significant differences were found in nearly 37% of participants, while improved and worsened symptoms were reported in 27.38% and 10.71% of participants, respectively; these data are similar to those reported in the relevant literature, since the great variety of disciplines and exercise programs used make it difficult to unify criteria that allow clarifying the role of exercise in PD [21]. However, the review by Armor et al., noted that regular physical activity lasting 45–60 min can provide beneficial effects [85].

Almost 65% of the participants in our study required treatment to relieve menstrual symptoms. The most popular treatment were pharmaceuticals, either on their own or combined with thermotherapy (i.e., the application of heat to the affected area). These data are consistent with those reported in the relevant literature [2,9,10,20,68,86,87,88]. However, one of the treatments most commonly described and used for the treatment of PD is the use of contraceptives, which, in our case, was not included as a form of treatment, since it was part of one of the variables used to classify the participants in our study. Despite being a form of treatment, and helping to improve symptoms, there were women who were recruited in the group of menstrual pain and contraceptive treatment, which indicates that its use does not completely eliminate menstrual pain in all cases. Something that could be assessed in future studies is the type of contraceptive treatment, because even though the vast majority of studies refer to combined oral contraceptives (COCs) for the treatment of PD, not all of the women in our study who were receiving contraceptive treatment were taking COCs; in some cases, they used an intrauterine device (IUD) or vaginal ring. Although the extant literature contends that all three forms of contraceptive treatment cause changes in menstrual flow and pain—and can even affect other symptoms associated with menstruation [2,8,9,10,20,89,90,91,92,93]—no studies to date have compared contraceptive use with PD. Unlike our research, in the vast majority of reviewed studies on PD, contraceptive treatment was considered an exclusion criterion.

### 4.2. Menstrual Pain and Severity

In addition to the lack of available data about the perception of physical pain in women with PD, we also found differences in the classification of dysmenorrhea based on the severity of their pain or symptoms, creating a very heterogeneous group in relation to the classification model and clinical criteria. In the case of our sample, the classification was made only using the VAS, considering women with PD who scored 3/10 on the VAS, as described by Huang et al. [23] and Gaubeca et al. [22].

The most subjective data on pain (i.e., the sensory, affective, and evaluative sphere), which we collected through the McGill Pain Questionnaire, revealed higher scores in the groups of women with PD; among this group, the highest scores were higher in the subgroup of women with PD who were not receiving contraceptive treatment. We classified the participants based on these data because, although the McGill Pain Questionnaire has been used in other studies on menstrual pain, it has not to date been used to make a classification based on severity.

### 4.3. Quality of Life

The results of our study indicate that women with PD and who are not receiving contraceptive treatment suffer from decreased quality of life during the menstrual period, especially with regard to physical function. This finding is in line with those of studies about the effects of menstruation [6,94,95].

### 4.4. Pain Mechanosensitivity

The main hypothesis of our study was that women with PD would have greater mechanosensitivity than women without PD; however, we did not find significant differences in terms of the decrease in PPT between women with and without dysmenorrhea, except in the measurement made in the right arm, in the luteal phase, between the groups that were under contraceptive treatment with and without PD; and in the left thigh, in the mid-cycle assessment, between the group of women without PD and who were under contraceptive treatment, compared with the two groups that did have PD, both with and without contraceptive treatment. The results differ from those presented in the study by Bajaj et al., who found differences in pressure between dysmenorrheic women and those who were not, even though we used the same anatomical references for our assessment [96]. Unlike our case, in their study, a prior assessment was carried out on all of the participants so that they knew how to indicate the moment of pressure pain. Such training was not carried out in our study, which may have influenced the sensations of perception of the patients. According to Vatine et al., PPT results can be influenced by mood, personality, the interpretation by participants of the instructions, familiarity with the technique, and differences in somatic sensitivity to pain [97]; these may have led to differences between our results and those of Bajaj et al., In addition, their sample comprised nursing and physiotherapy students exclusively, unlike our more heterogeneous sample, and their sample had a higher average age. This mean age of the study patients in the case of Bajaj et al., was 25.5 and 28 years for women with and without PD, respectively [96]. A higher age was found in our study, reaching a mean of 30.71 years for the group of women with PD and 35.47 years for the group of women without PD, who were not under contraceptive treatment. In the study by Bajaj et al., other types of measurements were made at the same points as the PPT, on sensitivity to heat, pressure in a pincer, and tactile stimulation. The assessment of algometry was continuous with that of a pincer in a subcutaneous fold of the same area [96], which could have increased the perception of pain compared with our study. Furthermore, women receiving contraceptive treatment were excluded from their study, but included in ours.

However, our results that coincide with those of Bajaj et al., are from the comparison between the different phases of the menstrual cycle, with the PPT being lower at the menstrual time-point vs. other time-points [96]; however, this was only found in the group of women with PD. In contrast, we found this result within all groups.

By comparison, Amodei and Nelson found no significant differences in pain thresholds and tolerance levels of dysmenorrheic and non-dysmenorrheic women at different phases of the menstrual cycle. They suggest that, although dysmenorrheic women report pain and distress throughout the menstrual and premenstrual phases of their cycles, these symptoms are more intense than those without, and there is no significant difference in their demonstrated ability to tolerate or cope with physical pain [98]. However, Tassorelli et al., suggested, based on their results, that women tended to have a greater susceptibility to the perception of pain symptoms during the luteal phase than during the follicular phase, which is probably the result of complex central and peripheral interactions between specific neurotransmitters, such as serotonin, opiates, and ovarian steroids [99].

We must also highlight that, although in our study three measurements were made on each of the assessment points, which were muscular structures, the measurement areas with algometry have generated discussion. This is because it is considered that the sensitivity to pressure measured with algometry varies according to the muscle and its location. Differences are also shown between the measurement of bone, neural, and muscular structures, with the PPT being less and more unpleasant and painful at the neural and bone level, respectively [100,101]. In addition, a wide diversity in terms of algometry and the number of measurements to establish the PPT can be found, from authors contending that a first measurement can sensitize the measurement point [97,102], to others who affirm that what exists is an accommodation in the successive measurements [103]. Yet others consider that the most accurate measurement is the average between the second and the third measurement [104,105].

One of the striking findings of our study is that in the algometry performed in the lumbar area, no statistically significant differences emerged, either between groups or between groups, despite being one of the main regions described as a zone of referred pain of lower-back menstrual pain. This could be justified by the results of the study by As-Sanie et al., in which the PPT was assessed in women with CPP and endometriosis with and without menstrual pain. They found differences in the perception of pain compared to that of healthy women, but reported a greater sensitivity to pain in locations far from the pelvic area, with the idea that amplification of central pain may play a role in the development of CPP [106].

Moreover, in patients with CPP, Fenton et al., assessed myofascial pain in the abdominal wall using algometry, applying pressure to 14 different points on the abdominal wall and making a second measurement on the same points after infiltrating the MTrPs with a 10 mL solution of 0.5% bupivacaine; they found an improvement of 75% in the PPT after the intervention [107]. However, the study did not have a control group; thus, it can be understood that, since there were so many infiltrated points, and since it involved the infiltration of a local anesthetic, the effects could be similar in the population without CPP.

In the rest of the comparisons regarding the perception of pain between women with and without menstrual pain, we found different ways of measuring and disparate results. Giamberardino et al., compared women with and without dysmenorrhea in different phases of the menstrual cycle; however, we must bear in mind that, in this case, they did so through electrical stimulation of the skin, subcutaneous tissue, and muscle. The measurement zones were also similar to ours, since they were made in the abdomen, deltoids, and quadriceps. The results indicate greater sensitivity in dysmenorrheic women and, in the menstrual phase, in electrical stimulation at the dermal, subcutaneous, and muscular levels in the abdomen and extremities. However, changes were also found in women without dysmenorrhea, in this case only at the muscular and abdominal subcutaneous levels [108]; this showed greater coincidence with our results, since the measurement was in muscular structures. In the study by Granot et al., women with dysmenorrhea had a greater perception of pain compared to women without dysmenorrhea, where the perception of pain was measured through evoked potentials [109].

Costantin et al., also measured pain thresholds via electrical stimulation and performed MTrP treatment at the lumbar level by local anesthetic infiltration. The results of the study show that, in patients with dysmenorrhea plus previous urinary calculosis with residual lumbar MTrPs, whose compression reproduces the typical pattern of urinary pain, viscerovisceral hyperalgesia persists despite not showing new calculosis. In fact, patients continue to experience more menstrual pain/muscle hyperalgesia referred from the uterus than patients with dysmenorrhea alone. Inactivation of lumbar MTrPs through local anesthetic injection results in a significant improvement in menstrual pain and referred muscle hyperalgesia of the uterus, in contrast to no improvement in the same parameters when there was no MTrP treatment [110]. This viscerovisceral hyperalgesia could explain the significant differences obtained in our study in the intragroup PPT measurements between the different phases of the menstrual cycle, since hyperalgesia or changes at the uterine level could affect the perception of pain and variations in mechanosensitivity [110].

Therefore, more in-depth future analysis is warranted, since there are other studies in which measurements have been made to assess sensitivity to pain in other musculoskeletal disorders, but taking into account the menstrual cycle. This was undertaken in the study by Isselee et al., in temporomandibular disorders, where it was observed that the PPTs are lower in the perimenstrual phase, and that they increase significantly and progressively in the follicular and luteal phases [111]. Therefore, these hormonal variations may be important when interpreting the results.

By comparison, the use of exogenous hormones can attenuate the effects of the cycle on experimental pain [112]. Therefore, studies such as ours that include a treatment group receiving contraceptive treatment should report on the type and dosage of that contraceptive [112]. However, a study by Isselee et al., showed that the PPTs of all the muscles evaluated were significantly lower in the perimenstrual phases in women both without contraceptive treatment and those taking COCs [102].

Payne et al., found increased pain sensitivity in adolescent girls and young adult women with PD at all phases of the menstrual cycle, consistent with evidence of central sensitization. However, measures of excitatory and inhibitory pain processing revealed no difference between groups [113]. The presence of generalized sensitivity to pressure pain is considered a manifestation of central sensitization, assuming the assessment areas distant from the area of clinical manifestation are asymptomatic and normal.

Therefore, further studies are required to determine whether women with dysmenorrhea are more susceptible to pain in general and, therefore, more likely to perceive dysmenorrhea; whether dysmenorrhea leads to a general lowering of the pain threshold, so that women who suffer from it are secondarily more susceptible to pain; or whether dysmenorrhea affects all women, because it varies depending on the menstrual cycle and its hormonal variations in the different phases of the cycle.

### 4.5. Myofascial Trigger Points in Primary Dysmenorrhea

This study found that local and referred pain caused by active MTrPs of the explored musculature of the abdomen, lower back, lower limb, and PF reproduced pain symptoms not only in PD patients, but also caused sensations similar to those felt during menstruation in women who did not have PD.

Despite the lack of previous research assessing the prevalence or relationship of MTrPs with PD, there are studies assessing this relationship with MTrPs of the abdominal muscles, finding that treatment by infiltration with lidocaine or dry needling provides benefits to these women [22,23,24]. One study included dry needling treatment of the hip muscles and manual therapy for the PF muscles [24]. The present study is the first to identify a direct relationship between MTrPs of the PF musculature in this patient profile.

Although the presence of MTrPs with PD has been little studied, we did find studies of the relationship of abdominal and pelvic MTrPs in women with CPP and with biopsy-confirmed endometriosis, which is relatively common. These studies also indicated that women with MTrPs were more likely to present signs of central sensitization [114].

MTrPs can become a self-sustaining source of pain, even after the visceral injury has resolved [115]. Active MTrPs, in particular, serve as a source of ongoing nociception, and can lower pain thresholds and increase visceral pain, referred pain, and central sensitization [35]. Aredo et al., suggest that, in the case of dysmenorrhea secondary to endometriosis, MTrPs that develop secondarily to the disease can maintain pain and dysfunction despite removal of the endometrial lesion and hormonal management [42]. Some studies promote the effectiveness of these techniques in the treatment of myofascial pelvic pain, such as Pastore et al. [116] and Bedaiwy et al., whose study showed a high prevalence of myofascial pain related to CPP. In their study, improvements were achieved in up to 63% of patients with CPP via physiotherapy treatment of the PF muscles. Improvements have been reported in terms of pain, and in terms of functionality [117], as shown by Espinosa et al., with its treatment in the PD [24].

Our study found a high presence of MTrPs in the abdominal muscles during menstruation, with higher prevalence of active MTrPs in the case of women with dysmenorrhea, and a higher prevalence of latent MTrPs in the case of women without PD for the rectus abdominis muscle, or oblique musculature, in all women. These MTrPs decrease during the periovulatory or intermenstrual phase, with the presence of latent MTrPs becoming greater than that of active MTrPs in the case of women with PD. This relationship with the abdominal muscles is not only present in PD, but also in other pathologies such as CPP, interstitial cystitis, or chronic prostatitis [45,49,50,56]. Montenegro et al., described that, in 15% of cases, CPP is associated with abdominal MPS, which is characterized by deep, intense pain in the abdominal region and generally affects women more than men [44]. In addition, the treatment of MTrPs in the abdominal wall was found to be effective in patients with CPP, although treatment by direct injection with lidocaine provided a clinically superior response to physical therapy in the long term [118].

The highest prevalence in our study of MTrPs related to PD was found in the muscles assessed by intrapelvic examination; a high frequency was found not only in the levator ani muscle, but also in the internal obturator and piriformis muscles. Although present in all exploration groups during menstruation, the rate of active MTrPs was higher in the PD groups, while the rate of latent MTrPs was higher in the non-PD groups. In the periovulatory or intermenstrual assessment, a similar pattern was found as that in the abdominal muscles. The absolute frequency of MTrPs was lower, and the number of active MTrPs was also higher, in proportion, than that of the latent MTrPs. These results align with those of Sedighimehr et al., who found that patients with CPP exhibited more tenderness in the levator ani, piriformis, and internal obturator muscles. They also indicated that more than 70% of the women in their study had dysmenorrhea [119]. Bassaly et al., also found a significant presence of MTrPs in the levator and obturator internus in women with interstitial cystitis, with a higher number of MTrPs in women who had endometriosis. Howard also found that the obturator internus and levator ani are the muscles that most frequently harbor MTrPs, in addition to the coccygeus and external anal sphincter muscles [120]. The data on these last two muscles do not coincide with those of our study. The study of Anderson et al., shows that, despite describing the presence of MTrPs in the coccygeus and anal sphincter muscles in patients with chronic prostatitis, the prevalence rates are much lower than those of the levator ani muscle [45]. In general, more studies support the relationship between PF muscle dysfunction and CPP patients [121,122,123], in addition to the efficacy of treatment directed at these structures, which provides benefits, relieving pain in patients with CPP after infiltration of lidocaine in the levator ani muscle; for example, the study of Langford et al. [60].

There is a greater number of latent MTrPs during menstruation in muscles that usually have MTrPs within the groups with menstrual pain compared to the groups without pain. This coincides with other studies of myofascial pain in other regions of the body, such as the lateral epicondylalgia [124], plantar fasciitis [125], or chronic lower-back pain [126]. However, we also found opposite data, in which the presence of latent MTrPs is similar in the pain groups compared to the control groups, as is the case of post-menisectomy pain [37] or mechanical neck pain [127]. These results are more consistent with those we found in the comparison between groups in the periovulatory or intermenstrual phase. These discrepancies could be due to the specific areas or muscles explored; in our case, they could be related to hormone levels and visceral referred pain, a hypothesis that we cannot confirm with current data.

Despite the clinical importance described by Shah et al., of active vs. latent MTrPs [128,129], the clinical relevance of latent MTrPs has increased in recent years [130,131], as both alter normal patterns of motor recruitment and movement efficiency, and induce mechanisms of peripheral and central sensitization [36,132,133]. Similar data were obtained by Sedighimehr et al., who found a significant reduction in mean PF muscle strength and endurance in women with CPP compared to those without [119]. This finding led us to consider the possible relevance of these latent MTrPs during the periovulatory or intermenstrual period as precursors of active MTrPs during the menstrual phase. In this way, the importance and additional contribution of MTrPs to the development and perpetuation of PD as a CPP entity could be explained in association with their involvement in peripheral and central sensitization, in addition to their possible bidirectional relationship with central sensitization. Its deactivation could be a relevant aspect to reverse central sensitization and improve associated pain.

In our study, as in the studies described above that found efficacy for PD through the treatment of MTrPs [22,23,24], the diagnostic criteria for MTrPs used were those established by the expert consensus of the Delphi panel in 2018, which are the presence of a taut band, a tender point, and referred pain [33]. However, other studies, such as the study by Itza et al., which explored the relationship of the CPP with the MTrPs of the PF, showed that gyro-amplitude electromyography is a reliable diagnostic test for detecting MPS of the PF musculature. They estimated the sensitivity (83%), specificity (100%), and positive (95% CI 1.00–1.00) and negative (95% CI 0.77–0.93) predictive values of the test; these findings are important because they support a diagnosis of PF MPS, although they offer insufficient predictive power to constitute it as a diagnostic test in patients with CPP [134]. In this regard, however, Hubbard and Berkoff described a non-specific electrodiagnostic version of MPS, describing electrical activity as a characteristic common to all MTrPs, in which the potentials were characterized by a high-frequency peak [135]. Previously, Simons et al., described low-amplitude motor endplate noise associated with the presence of MTrPs, considering it spontaneous electrical activity [136]. Furthermore, Partanen et al., described that taut bands may be local contraction points in skeletal motor units, which could be caused by a sustained reflex drive in muscle spindles. On electromyography, this can be seen as complex repetitive discharges; therefore, we can deduce that MTrPs are related to increased excitability of muscle spindles [137]. We can only mention this as a future line of research that will help in the earlier detection of the presence of MPS in PF musculature, since there is little evidence available on its presence and the pathology of chronic pelvic pain, including PD.

### 4.6. Referred Pain Areas

As mentioned above, the presence of MTrPs with PD has been little studied. Less valued, however, is the relationship between the areas of referred pain of MTrPs and menstrual pain, despite the fact that the areas usually described as experiencing menstrual pain are within the referred pain maps of muscles such as the rectus abdominis, external oblique, gluteus, adductor magnus, piriformis, obturator internus, and PF musculature. In this sense, this study is the first to find a relationship between the presence of MTrPs—and their areas of referred pain—with the pain suffered by women with PD. Our study also reproduced the characteristic clinical picture of this pelvic alteration.

The results obtained in our study allow expansion of the referred pain reference maps of the MTrPs of the studied musculature reproduced both at the menstrual moment and in the periovulatory or intermenstrual phase. We found that, although the representation is greater in the women with PD, there was also a similar reality in women without dysmenorrhea. We found that the gluteal musculature extends its referred pain pattern to the abdominal area and anterior pelvic structures such as the pubis, groin, and vulva. In the case of the quadratus lumborum and the abdominal muscles, we found that the abdominal surface in which the referred pain is caused increases with respect to the areas already described by Travell and Simons. The most striking and relevant data that we obtained are the areas of referred pain of the PF muscles and the piriformis and internal obturator muscles. In addition to relating the presence of MTrPs in these muscles within highly restricted areas at the level of the gluteus, sacrum, and coccyx, we can also include as areas of referred pain the abdominal area (mainly suprapubic area and flank); lumbar area; anterior, internal, and posterior parts of the thigh; groin; and genitourinary and vulvar areas. Within the available literature, in the case of the PF muscles, few studies have suggested patterns of referred pain that differed from those described by Travell and Simons [27,39], but not related to PD. In the study by Bassaly et al., an examination of the rectus abdominis, levator ani, and obturator internus muscles was performed in patients with interstitial cystitis. They found that the areas of pain or discomfort caused by MTrPs are projected towards the suprapubic, lumbar, anal, and vulvar regions. These results highlight that a closer relationship was detected with the symptomatology of the study; the MTrPs of the left side of the women were explored, although the relationship was found to be not significant [50]. The study by Anderson et al., had the same objective as ours, regarding the definition of areas of referred pain, but in men with chronic prostatitis [45]. The results coincide with our results and those found by Bassaly et al., highlighting the suprapubic and genital area, with the most prevalent rate of MTrPs being in the levator ani [45,50].

Although this type of exploration was not included in our study, we must consider that visceral structures can also cause referred pain, and their characteristic areas have also not been widely studied. In this regard, Arendt-Nielsen et al., found that women with dysmenorrhea had larger areas of referred pain compared to controls. Pain threshold at first stimulus was significantly higher in patients than that of controls, but significantly decreased when the cervix was repeatedly distended. For prolonged cervical distention, pain ratings increased significantly in women with dysmenorrhea but decreased in control women. This may mean that sensitization to pain by temporary summation (i.e., increased pain during prolonged stimulation) and the facilitation of areas of referred pain can be considered as indicators of changes in the central nervous system in women with dysmenorrhea. Referred pain caused by this cervical distention harbored the usual areas of menstrual pain: the lumbar region, abdominal region, pelvic region, and thighs [138].

### 4.7. Hormonal Influence of the Menstrual Cycle on the Perception of Pain

There is much controversy in the literature about whether the timing of the menstrual cycle has an effect on pain perception in women. A review by Iacovides et al., reported that most well-controlled studies found that the phase of the menstrual cycle had no effect on pain perception in healthy, pain-free women. However, these results may be surprising considering the fluctuations in reproductive hormones throughout the cycle, and considering that animal studies have shown that progesterone and estradiol influence the response to pain. Some imaging studies show that there are, indeed, differences in patterns of brain activation in regions involved in cognitive modulation of pain in association with hormonal changes in the menstrual cycle, even when behavioral responses to pain have not changed [139]. When other pain conditions are considered, as in the study by LeResche et al., examining temporomandibular disorders, the results obtained suggest that temporomandibular pain in women is higher when estrogen levels are low, although the rapid variation in these levels may also be associated with increased pain [140].

The assessments in our study were made based on the menstrual calendar during menstruation, the periovulatory or intermenstrual phase, and the luteal phase. As with other studies, we assessed differences in hormone levels across these phases [98,108]. However, Sherman et al., suggest that the main limitation of much previous research on the menstrual cycle is that the underlying objective has been to detect relationships between pain and hormones, in the absence of a direct measurement of hormone levels, since the phases of the menstrual cycle have been taken as a reference. Regarding the menstrual cycle, there is significant heterogeneity between the studies when considering the phase and its recognition in the population of women assessed, regardless of the variability in both the length of the menstrual cycle and ovulation, and considering that approximately 20% of cycles may be anovulatory [112].

Bartley et al., assessed differences in perception of mechanical, electrical, and ischemic pain by measuring the tolerance and pain threshold in healthy women in the middle follicular and late luteal phases. They established minimal variations in pain sensitivity and spinal nociceptive processing between the phases, which would imply that the hormonal variation between these two phases has a minimal effect on the response to pain [141].

However, the latest recommendations on the considerations of the menstrual cycle were determined by the type of study and its variables, considering that in cross-sectional studies, the menstrual cycle is not the main variable of interest, although its potential effects should be controlled for the main variables. As is the case in our study, it would not be necessary to make such an exhaustive hormonal registry. The selection of the phase could be adjusted according to the menstrual calendar, regarding women with regular menstrual cycles [142].

MacLean and Hayashi explain that menstrual cycles, which are regulated by complex hormonal mechanisms, repeat and alter the local endometrial environment. Thus, the local hormonal environment cannot be precisely defined in each cycle. They note that some gynecological disorders may be due to disrupted hormone production, progesterone resistance, altered hormone-dependent gene expression, common somatic genetic mutations, and/or side effects of hormonal treatments, and many of these patients suffer from CPP and/or dysmenorrhea, which reduces the quality of life [143].

Pelvic pain is recognized as being driven by hormones, for example, endometriosis; thus, the role of these substances should be considered. In addition, there is considerable overlap between conditions that can cause chronic pelvic pain (endometriosis, interstitial cystitis, and irritable bowel syndrome), making it difficult to definitively assign causality to any one of them [144].

The study by Rezaii et al., showed more effective pain modulation in the ovulatory phase of the menstrual cycle, when estradiol levels are higher and progesterone levels are lower, than in the early follicular phase, when levels of both hormones are low, as measured by pressure tolerance to pain in healthy women. In conclusion, the results of this study showed more effective pain modulation in the ovulatory phase of the menstrual cycle, when estradiol levels are high and progesterone levels are low, than in the early follicular phase, when estradiol and progesterone levels are low [145].

One must ask whether the absolute level or the variation in hormonal levels is more important, and we must consider that most of the studies recorded estrogens levels rather than progestogen levels [144]. According to Christin-Maitre, in the available studies on the menstrual cycle, menstruation, and dysmenorrhea, ample reference can be found to menstruation as a psychosocial phenomenon strongly affected by historical evolution and cultural factors. In many women it is considered an unknown process until the moment of their “suffering”; thus, it can be strongly influenced by the environment, and by external factors such as stress, intense physical activity, and weight extremes, which can alter normal hypothalamic secretion of GnRH [146].

It is tempting to assume that finding an association between an outcome and a particular phase of the cycle implies that concurrent hormonal events drive symptom change. However, at least with respect to premenstrual pain syndrome, studies suggest delayed effects of hormonal change, such that the onset of symptoms occurs at a different phase than the hormonal event [142].

For all these reasons, the phases or moments of measurement should be standardized. Alternatively, hormone tests and more frequent assessments can be conducted to establish a more direct relationship between the different variables with respect to hormone levels. The importance of understanding the hormonal flow and how it affects human physiological and psychosocial factors should be recognized.

### 4.8. Peripheral and Central Sensitization, and Their Relationship with Menstruation and Primary Dysmenorrhea

One of our study’s objectives was to detect a generalized sensitivity of pain to pressure in women with PD. Therefore, we considered it a manifestation of the central sensitization that these women could experience compared with women without menstrual pain. However, the data we obtained on PPT between the different phases of the menstrual cycle led us to consider that there may not be an alteration in sensitivity exclusive to PD but, rather, generalized by the menstrual cycle. According to Greenwald and Shafritz, the lowest PPT found in symptomatic areas, such as the abdomen in our study, would represent primary hyperalgesia due to sensitized polymodal nociceptors within the injured musculoskeletal structures related to peripheral sensitization. In addition, the lower PPT in the extremities in our study implies the presence of secondary hyperalgesia, with generalized hyperexcitability of the central nociceptive pathways related to central sensitization [147].

We must also consider the role of MTrPs in peripheral and central sensitization, and consider the high number of both active and latent MTrPs in some muscles in our research, since their presence may contribute to exacerbating and perpetuating pain in women with PD. In line with our results, Wang et al., concluded that MTrPs were associated with an early manifestation of local and generalized mechanical hyperalgesia that contributes to peripheral and central sensitization, which favors the spatial spread of mechanical pain [148]. Furthermore, MTrPs can be the generators of nociceptive impulses that lead to the process of generalized hyperalgesia and central sensitization [133,149].

Central sensitization, including lower PPT and extension of pain areas, may contribute to increased MTrPs [35], in the same way that active MTrPs may contribute to the spatial spread of pain and/or generalized pain in conditions of musculoskeletal pain [150]. In addition, this hyperalgesia can favor the presence of more active MTrPs in the same muscle [151] or in several functionally related muscles in situations of chronic musculoskeletal pain [118].

The sustained nociceptive stimuli that can cause hyperirritability of the MTrPs of a muscle can cause the secretion of sensitizing substances such as PGs, among others, which lower the activation threshold of the neuron such that they sensitize nociceptors [36,152]. These are PGs, which are also considered the main cause of the etiopathogenesis of menstrual pain [5,9,10,11,12,80].

We can also see differences between genders in terms of pain perception; more specifically, we found that a greater sensitivity to pain and risk of clinical pain was more common in women. In addition, although the specific etiology underlying these sex differences is unknown, it is highly likely that multiple biological and psychosocial processes are contributing factors [153]. Pieretti et al., highlight the importance of the interaction of the opioidergic system with sex hormones [154].

There are two aspects of central sensitization that are especially relevant for women with CPP: viscerosomatic convergence and viscerosomatic reflex [42]. Almost all spinal neurons that receive visceral input also receive somatosensory input from muscle and skin through a process known as viscerosomatic convergence [155]. Input convergence makes precise localization and discrimination of sensory information difficult [156]. This is also the basis of referred pain and explains why visceral pathologies are commonly felt as pain in somatic structures—particularly muscles—innervated by the same spinal segment; in addition, since visceral afferent fibers terminate in several spinal segments above and below the entry level of the segment, referred pain may be present in areas remote from the affected visceral organ [42,155]. Therefore, this viscerosomatic convergence would explain how noxious visceral input can sensitize multiple areas of the spinal cord, generating the broad areas of allodynia, hyperalgesia, and referred pain seen with somatic dysfunction [42].

The data provided by Sánchez et al., recently demonstrated that somatovisceral reflexes involving the bladder, urethra, and musculature of PF are sensitive to ovarian hormones, and estrogens play an essential role in these reflexes. Their findings support the notion that alterations in the activity of the pubococcygeus muscle during urination are sensitive to estrogens. This highlights the need to establish a more direct relationship between muscle assessment and hormone levels, which will depend on the moment of the menstrual cycle in which it occurs [157]. Prendergast and Weiss suggest that chronic pain caused by viscerovisceral and somatovisceral reflexes could be an indirect mechanism involved in interstitial cystitis that is accompanied by painful spasms of the PF musculature [158].

Viscerosomatic convergence may not only provide the means for pain referral to somatic structures, but also govern the reflex that induces muscle spasm and eventual MTrP formation. In turn, the MTrPs can serve as an additional source of nociceptive information and become a key component of the CPP. Therefore, its inhibition could reverse central sensitization and improve the pain associated with endometriosis [42]. Viscerovisceral hyperalgesia between the uterus and urinary tract may persist after stone passage due to nociceptive inputs of MTrPs into the referred urinary area, since MTrP treatment effectively reverses intensified menstrual symptoms [110].

Central sensitization and myofascial pain secondary to active MTrPs are likely other sources of pain initiation, amplification, and perpetuation. Either could easily spread pain-related symptoms in women, even after surgical and medical/hormonal treatment for endometriosis has been optimized. Unfortunately, both central sensitization and myofascial dysfunction are frequently overlooked in the evaluation, diagnosis, and treatment of CPP associated with endometriosis [42].

Tu et al., noted that excessive excitatory input during menstrual pain can induce a compensatory inhibitory mechanism in several somatic sensorimotor regions [159]. In addition, like Vincent et al., they considered that constant and repeated stimulation of nociceptive pathways, as in the case of menstrual pain, can give rise to functional and structural alterations of the CNS, thereby giving rise to central sensitization [159,160]. In the same regard, Iacovides et al., concluded that limiting cyclical noxious input to the central nervous system in women with severe dysmenorrhea via analgesic treatment could reduce the chances of developing hyperalgesia and possibly other chronic pain conditions, as dysmenorrhea predisposes women to diffuse muscle hyperalgesia and clinically relevant deep muscle pain [161].

It is important to consider CPP associated with dysmenorrhea secondary to endometriosis from a global pain-focused perspective, as MTrPs and central sensitization appear to contribute significantly to clinical manifestations. Although the direct innervation of endometrial lesions may set the stage for visceral nociception and peripheral sensitization, over time central sensitization creates a process for pain maintenance that is independent of the initial pathology and is potentially reversible [115].

Oladosu et al., demonstrated the association of menstrual pain with abnormal autonomic activity and bladder sensitivity, even 2 weeks after menstruation. Both dysmenorrheic and bladder pain syndrome participants reported increased sensitivity to bladder pain compared to controls, with frequent comorbidity [162].

### 4.9. Future Lines of Research

Due to the large amount of data collected in this study and the controversial results found in existing studies, more research is required to clarify whether the relationship between MPS and PD is clinically relevant. If so, it may offer new therapeutic approaches for women with PD whose quality of life is affected monthly, and who resign themselves to the use of pharmacological treatments on a regular basis.

Our study is observational in nature and relied on a convenience sample. As such, clinical trials with randomized procedures are encouraged, as they may provide additional important data about women with PD.

Furthermore, although there is a high correlation between the summary measures of physical health and mental health between the SF-12 and the SF-36 data, it would have been convenient to use the SF-36 to avoid a loss of precision in the scores given our small sample size.

Our participants were also not classified based on the severity of their dysmenorrhea, which could have helped us determine the relevance of the study variables according to their degree of severity. Moreover, classifications did not consider the type of contraceptive treatment used.

Despite complying with the recommendations on the choice of menstrual phases (according to the menstrual calendar), using hormonal controls through urine or saliva tests could have allowed us to establish a more direct relationship between our results and hormonal levels.

In addition, exploration by palpation of some study muscles may not be completely reliable because the muscles we studied are found in deeper planes, which may undermine the accuracy of our conclusions regarding the direct relationship indicated by the results, or relate these to more superficial muscle planes. Thus, as in the case of the subregions of the levator ani, it is difficult to ensure that the identified referred pain originated exclusively from that portion. Furthermore, despite our awareness of the fact that the most anterior and most posterior fibers do correspond to the so-called bundle, it would be convenient to encompass the referred pain patterns of the puborectalis, pubococcygeus, and iliococcygeus muscles into one pattern.

Finally, despite having made a very detailed and specific percentage record of referred pain areas, validated software used either at the time of recording the pain areas or during analysis would have allowed us to make a real representation of the pain maps instead of creating a graphical illustration based on the data.

## 5. Conclusions

We conclude that there are few differences in musculoskeletal mechanosensitivity between women with and without PD, except in the luteal phase in women with PD who are under contraceptive treatment compared to those who, while under contraceptive treatment, do not present PD, in dermatomes C7-C8; and in the ovulatory phase in women with PD compared to those who do not have PD, but who are under contraceptive treatment, in dermatomes L2-L3. Furthermore, we conclude that there are bilateral differences in musculoskeletal mechanosensitivity in dermatomes C7-C8, L2-L3, and T10-T12, but there are no differences in dermatomes S2-S4 between the different phases of the menstrual cycle. However, a lower PPT in the menstrual phase was found, which was more pronounced in some locations in women receiving contraceptive treatment.

A higher prevalence of active MTrPs was found in the rectus abdominis, gluteus maximus, ischiocavernosus, and pubococcygeus muscles during the menstrual phase. A higher prevalence of active MTrPs in the iliococcygeus muscle and latent MTrPs in the ischiocavernosus muscle was found in the menstrual, periovulatory, and intermenstrual phases in women with PD. We also highlight that, during the menstrual phase, active MTrPs in the iliococcygeus muscle are common, being present in more than 50% of women in all experimental groups, in more than 70% of women with PD, and in almost all women who were not under contraceptive treatment. The internal obturator muscle presented MTrPs, whether active or latent, during the menstrual phase in all of our participants.

The areas of referred pain in the studied muscles, especially in the PF muscles, were found to be greatly extended relative to previous conceptions, including abdominal areas and the lower extremities. At the time of menstruation, the inguinal area was referred to with the greatest significance by women with PD among the abdominal muscles and the levator ani muscle. Outside of menstruation, the areas in which the pain was significantly referred were the hypogastrium, the vulva and the rectum, and the anus, from the iliococcygeus, obturator internus, and puborectalis muscles, respectively.

## Figures and Tables

**Table 1:** Demographics.

	Total	NDNC	NDWC	WDNC	WDWC	*p*-Value * F (*η*^2^)
	Mean ± SD (99% CI, n = 84)	Mean ± SD (99% CI, n = 21)	Mean ± SD (99% CI, n = 21)	Mean ± SD (99% CI, n = 21)	Mean ± SD (99% CI, n = 21)	
Age (years)	31.20 ± 7.65 (28.99–33.40)	35.47 ± 9.36 (29.65–41.29)	30.57 ± 5.57 (27.11–34.03)	30.71 ± 7.65 (25.96–35.46)	28.04 ± 5.97 (28.99–33.40)	*p* = 0.013 F = 3.792 *η^2^ = 0.124*
Weight (kg)	58.22 ± 6.50 (56.35–60.09)	61.08 ± 8.42 (55.84–66.31)	57.23 ± 4.04 (54.72–59.75)	56.80 ± 5.68 (53.27–60.34)	57.76 ± 6.60 (53.65–61.86)	*p* = 0.129 F = 1.944 *η^2^ = 0.068*
Height (m)	1.64 ± 0.04 (1.63–1.65)	1.64 ± 0.04 (1.61–1.67)	1.65 ± 0.03 (1.63–1.67)	1.63 ± 0.03 (1.60–1.65)	1.65 ± 0.05 (1.61–1.68)	*p=* 0.334 F = 1.151 *η^2^ = 0.041*
BMI (Kg/m^2^)	21.51 ± 2.25 (20.87–22.16)	22.61 ± 3.26 20.59–24.64)	20.93 ± 1.79 (19.82–22.05)	21.31 ± 1.28 (20.52–22.11)	21.20 ± 1.96 (19.98–22.42)	*p=* 0.070 F = 2.441 *η^2^ = 0.084*

Abbreviations: BMI: body mass index. NDNC: non-dysmenorrhea non-contraceptives group; NDWC: non-dysmenorrhea, with contraceptives group; WDNC: with dysmenorrhea non-contraceptives group; WDWC: with dysmenorrhea with contraceptives group. SD: standard deviation; A *p* < 0.01 with a 99% confidence interval was considered statistically significant. * One-way ANOVA was used.

**Table 2:** Qualitative clinical descriptive data.

		Total (n = 84)	NDNC (n = 21)	NDWC (n = 21)	WDNC (n = 21)	WDWC (n = 21)	*p*-Value * χ^2^
		n (%)	n (%)	n (%)	n (%)	n (%)	
Contraceptive treatment	YES	42 (50)	0 (0)	21 (100)	0 (0)	21 (100)	***p* < 0.001** *χ*^2^ = 80.188
	NO	42 (50)	21 (100)	0 (0)	21 (100)	0 (0)	
Type of contraceptive treatment	NO TRT	42 (50)	21 (100)	0 (0)	21 (100)	0 (0)	***p* < 0.001** *χ*^2^ = 83.765
	RING	11 (13.10)	0 (0)	7 (33.33)	0 (0)	4 (19.05)	
	IUD	6 (7.14)	0 (0)	2 (9.52)	0 (0)	4 (19.05)	
	COCs	25 (29.76)	0 (0)	12 (57.14)	0 (0)	13 (61.90)	
Low back pain	YES	42 (50)	9 (42.86)	9 (42.86)	16 (76.19)	8 (9.52)	*p* = 0.05 *χ*^2^ = 7.81
	NO	42 (50)	12 (57.14)	12 (57.14)	5 (23.81)	13 (61.90)	
Pelvic pain	YES	24 (28.57)	5 (23.81)	6 (7.14)	8 (9.52)	5 (23.81)	*p* = 0.706 *χ*^2^ = 1.400
	NO	60 (71.43)	16 (76.19)	15 (71.43)	13 (61.90)	16 (76.19)	
GI symptoms during menstruation	YES	53 (63.10)	15 (71.43)	7 (33.33)	17 (20.24)	14 (66.67)	*p* < 0.01 * χ^2^ = 11.606
	NO	31 (36.90)	6 (7.14)	14 (66.67)	4 (19.05)	7 (33.33)	
Common GI symptoms	YES	13 (15.48)	4 (19.05)	5 (23.81)	1 (4.76)	3 (14.28)	*p* = 0.364 *χ*^2^ = 3.185
	NO	71 (84.52)	17 (20.24)	16 (76.19)	20 (95.24)	18 (85.71)	
Dyspareunia	YES	25 (29.76)	6 (7.14)	7 (33.33)	6 (7.14)	6 (7.14)	*p* = 0.982 *χ*^2^ = 0.171
	NO	59 (70.24)	15 (71.43)	14 (66.67)	15 (71.43)	15 (71.43)	
Menstrual pain	YES	58 (69.05)	9 (42.86)	7 (33.33)	21 (100)	21 (100)	***p* < 0.001** * χ^2^ = 34.659
	NO	26 (30.95)	12 (57.14)	14 (66.67)	0 (0)	0 (0)	
Menstrual Pain Treatment	Heat	2 (2.38)	0 (0)	1 (4.76)	1 (4.76)	0 (0)	*p* < 0.144 *χ*^2^ = 17.147
	Medication	26 (30.95)	7 (33.33)	3 (14.28)	8 (9.52)	8 (9.52)	
	Heat and medication	24 (28.57)	2 (9.52)	1 (4.76)	10 (47.62)	11 (52.38)	
	Kinesiotape	2 (2.38)	0 (0)	1 (4.76)	0 (0)	1 (4.76)	

Abbreviations: GI: gastrointestinal; NDNC: non-dysmenorrhea non-contraceptive group; NDWC: non-dysmenorrhea with contraceptives group; WDNC: with dysmenorrhea non-contraceptives group; WDWC: with dysmenorrhea with contraceptives group. A *p* < 0.01 (**Bold**) was considered statistically significant. * One-way ANOVA was used.

**Table 3:** Questionnaire SF-12.

	Total	NDNC	NDWC	WDNC	WDWC	*p*-Value * F (*η*^2^)	Post-Hoc *P* (d Cohen) a-NDNC vs. NDWC b-NDNC vs. WDNC c-NDNC vs. WDWC d-NDWC vs. WDNC e-NDWC vs. WDWC f-WDNC vs. WDWC
	Mean ± SD (99% CI, n = 84)	Mean ± SD (99% CI, n = 21)	Mean ± SD (99% CI, n = 21)	Mean ± SD (99% CI, n = 21)	Mean ± SD (99% CI, n = 21)		
Physical Health	18.07 ± 1.81 (17.54–18.59)	18.09 ± 2.02 (16.83–19.35)	18.95 ± 1.20 (18.20–19.69)	16.85 ± 2.15 (15.52–18.19)	18.38 ± 1.02 (17.74–19.01)	*p* = 0.001 * F = 5.860 *η^2^ = 0.180*	a-*p* = 0.606 *d* = 0.517 b-*p* = 0.113 *d* = 0.594 c-*p* = 1.000 *d* = 0.181 d-***p* = 0.001** *d* = 1.206 e-*p* = 1.000 *d* = 0.511 f-*p* = 0.025 *d* = 0.909
Physical Health (%)	86.36 ± 12.98 (82.63–90.10)	86.52 ± 14.43 (77.55–95.48)	92.61 ± 8.58 (87.28–97.95)	77.71 ± 15.46 (68.11–87.31)	88.61 ± 7.28 (84.09–93.14)	*p* = 0.001 * F = 5.805 *η^2^ = 0.179*	a-*p* = 0.619 *d* = 0.513 b-*p* = 0.117 *d* = 0.589 c-*p* = 1.000 *d* = 0.182 d-***p* = 0.001** *d = 1.191* e-*p* = 1.000 *d* = 0.502 f-*p* = 0.025 *d* = 0.902
Mental Health	20.90 ± 3.13 (20.00–21.80)	20.04 ± 3.98 (17.57–22.51)	22.61 ± 2.13 (21.29–23.94)	20.38 ± 2.97 (18.53–22.22)	20.57 ± 2.71 (18.88–22.25)	*p* = 0.31 F = 3.105 *η^2^ = 0.104*	a-*p* = 0.044 *d* = 0.805 b-*p* = 1.000 *d* = 0.096 c-*p* = 1.000 *d* = 0.155 d-*p* = 0.113 *d* = 0.862 e-*p* = 0.187 *d* = 0.836 f-*p* = 1.000 *d* = 0.066
Mental Health (%)	71.00 ± 14.90 (66.71–75.28)	66.90 ± 18.88 (55.18–78.62)	79.19 ± 10.12 (72.90–85.47)	68.42 ± 14.04 (59.70–77.15)	69.47 ± 12.94 (61.43–77.51)	*p* = 0.029 F = 3.153 *η^2^ = 0.106*	a-*p* = 0.041 *d* = 0.811 b-*p* = 1.000 *d* = 0.091 c-*p* = 1.000 *d* = 0.158 d-*p* = 0.104 *d* = 0.880 e-*p =* 0.187 *d* = 0.836 f-*p* = 1.000 *d* = 0.077
Total	38.97 ± 3.92 (37.84–40.10)	38.14 ± 4.74 (35.19–41.08)	41.57 ± 2.80 (39.83–43.31)	37.23 ± 3.92 (34.80–39.67)	38.95 ± 2.65 (37.30–40.60)	*p* = 0.002 * F = 5.54 *η^2^ = 0.172*	a-*p* = 0.018 *d* = 0.881 b-*p* = 1.000 *d* = 0.209 c-*p* = 1.000 *d* = 0.210 d-***p* = 0.001** *d* = 1.274 e-*p* = 0.132 *d* = 0.961 f-*p* = 0.782 *d* = 0.514
Total (%)	77.05 ± 11.15 (73.84–80.26)	74.71 ± 13.57 (66.28–83.14)	84.38 ± 7.93 (79.45–89.30)	72.19 ± 11.14 (65.27–79.10)	76.95 ± 7.63 (72.21–81.69)	*p* = 0.002 * F = 5.396 *η^2^ = 0.168*	a-*p* = 0.020 *d* = 0.870 b-*p* = 1.000 *d* = 0.202 c-*p* = 1.000 *d* = 0.203 d-***p* = 0.002** *d* = 1.260 e-*p* = 0.137 *d* = 0.954 f-*p* = 0.843 *d* = 0.498

Abbreviations: NDNC: non-dysmenorrhea non-contraceptive group; NDWC: non-dysmenorrhea with contraceptives group; WDNC: with dysmenorrhea non-contraceptives group; WDWC: with dysmenorrhea with contraceptives group. SF-12: Short Form 12 Health Survey. SD: standard deviation; A *p* < 0.01 with a 99% confidence interval (**Bold**) was considered statistically significant. * One-way ANOVA was used.

**Table 4:** The McGill Pain Questionnaire.

	Total	NDNC	NDWC	WDNC	WDWC	*p*-Value * F (*η*^2^)	Post-Hoc *P* (d Cohen) a-NDNC vs. NDWC b-NDNC vs. WDNC c-NDNC vs. WDWC d-NDWC vs. WDNC e-NDWC vs. WDWC f-WDNC vs. WDWC
	Mean ± SD (99% CI, n = 84)	Mean ± SD (99% CI, n = 21)	Mean ± SD (99% CI, n = 21)	Mean ± SD (99% CI, n = 21)	Mean ± SD (99% CI, n = 21)		
PRI-S	14.92 ± 10.34 (11.95–17.90)	11.61 ± 8.89 (6.08–17.13)	5.61 ± 6.93 (1.31–9.92)	22.52 ± 9.02 (16.92–28.12)	19.95 ± 6.77 (15.74–24.15)	*p* = 0.000 * F = 19.879 *η^2^ = 0.427*	a-*p* = 0.102 *d* = 0.752 b-***p* = 0.000** *d* = 1.218 c-***p* = 0.007** *d* = 1.055 d-***p* = 0.000** *d* = 2.102 e-***p* = 0.000** *d* = 2.093 f-*p* = 1.000 *d* = 0.326
PRI-A	1.17 ± 1.48 (0.75–1.60)	0.76 ± 0.83 (0.24–1.27)	0.19 ± 0.40 (−0.05–0.44)	2.14 ± 1.87 (0.97–3.30)	1.61 ± 1.53 (0.66–2.57)	*p* = 0.000 * F = 9.462 *η^2^ = 0.262*	a-*p* = 0.944 *d* = 0.874 b-***p* = 0.005** *d* = 0.953 c-*p* = 0.212 *d* = 0.690 d-***p* = 0.000** *d* = 1.442 e-***p* = 0.004** *d* = 1.269 f-*p* = 1.000 *d* = 0.310
PRI-E	1.85 ± 1.28 (1.48–2.22)	1.61 ± 0.97 (1.01–2.22)	0.61 ± 0.86 (0.08–1.15)	2.95 ± 1.07 (2.28–3.61)	2.23 ± 0.94 (1.65–2.82)	*p* = 0.000 * F = 22.007 *η^2^ = 0.452*	a-***p* = 0.007** *d* = 1.090 b-***p* = 0.000** *d* = 1.312 c-*p* = 0.246 *d* = 0.649 d-***p* = 0.000** *d* = 2.410 e-***p* = 0.000** *d* = 1.798 f-*p* = 0.114 *d* = 0.714
PRI-Total	17.96 ± 12.34 (14.41–21.51)	14.09 ± 10.46 (7.59–20.59)	6.33 ± 7.85 (1.45–11.21)	27.47 ± 9.97 (21.28–33.66)	23.95 ± 8.43 (18.71–29.18)	*p* = 0.000 * F = 22.649 *η^2^ = 0.459*	a-*p* = 0.048 *d* = 0.839 b-***p* = 0.000** *d* = 1.309 c- ***p* = 0.005** *d* = 1.037 d-***p* = 0.000** *d* = 2.356 e-***p* = 0.000** *d* = 2.163 f-*p* = 1.000 *d* = 0.381
Nº of words	8.38 ± 4.61 (7.05–9.70)	7.19 ± 4.26 (4.54–9.83)	4.38 ± 4.34 (1.68–7.07)	11.09 ± 3.46 (8.94–13.24)	10.85 ± 2.63 (9.22–12.49)	*p* = 0.000 * F = 15.478 *η^2^ = 0.367*	a-*p* = 0.103 *d* = 0.653 b-***p* = 0.007** *d* = 1.004 c-***p* = 0.013** *d* = 1.033 d-***p* = 0.000** *d* = 1.709 e-***p* = 0.000** *d* = 1.803 f-*p* = 1.000 *d* = 0.078
PPI	1.63 ± 1.01 (1.33–1.92)	1.19 ± 0.51 (0.87–1.50)	0.90 ± 0.62 (0.51–1.29)	2.66 ± 1.15 (1.94–3.38)	1.76 ± 0.62 (1.37–2.14)	*p* = 0.000 * F = 21.343 *η^2^ = 0.445*	a-*p* = 1.000 *d* = 0.510 b-***p* = 0.000** *d* = 1.652 c-*p* = 0.112 *d* = 1.004 d-***p* = 0.000** *d* = 1.905 e-***p* = 0.003** *d* = 1.387 f-***p* = 0.002** *d* = 0.974

Abbreviations: NDNC: non-dysmenorrhea non-contraceptive group; NDWC: non-dysmenorrhea with contraceptives group; PPI: Pain Intensity Index; PRI: Pain Assessment Index; PRI-A: Pain Rating Index–Affective; PRI-E: Pain Assessment Index–Evaluative; PRI-S: Pain Rating Index–Sensory; WDNC: with dysmenorrhea non-contraceptives group; WDWC: with dysmenorrhea with contraceptives group. SD: standard deviation; A *p* < 0.01 with a 99% confidence interval (**Bold**) was considered statistically significant. * One-way ANOVA was used.

**Table 5:** Presence of MTrPs in the external musculature evaluated in the menstrual phase.

Muscles	Presence of MTrPs	Total (n = 84)	NDNC (n = 21)	NDWC (n = 21)	WDNC (n = 21)	WDWC (n = 21)	*p*-Value * χ^2^
		n (%)	n (%)	n (%)	n (%)	n (%)	
Rectus abdominis	Does not have	7 (8.33)	4 (19.04)	3 (14.28)	0	0	*p* < 0.001 * χ^2^ = 25.510
	Active	28 (33.33)	2 (9.52)	2 (9.52)	11 (52.38)	13 (61.90)	
	Latent	49 (58.33)	15 (71.42)	16 (76.19)	10 (47.61)	8 (38.09)	
External oblique	Does not have	13 (15.47)	6 (28.57)	4 (19.04)	2 (9.52)	1 (4.76)	*p* = 0.082 *χ*^2^ = 11.230
	Active	10 (11.90)	3 (14.28)	0	5 (23.80)	2 (9.52)	
	Latent	61 (72.61)	12 (57.14)	17 (80.95)	14 (66.66)	18 (85.71)	
Internal oblique	Does not have	34 (40.47)	13 (61.90)	11 (52.38)	3 (14.28)	7 (33.33)	*p* = 0.015 *χ*^2^ = 15.827
	Active	23 (27.38)	1 (4.76)	4 (19.04)	10 (47.61)	8 (38.09)	
	Latent	27 (32.14)	7 (33.33)	6 (28.57)	8 (38.09)	6 (28.57)	
Adductor magnus	Does not have	6 (7.14)	3 (14.28)	0	1 (4.76)	2 (9.52)	*p* = 0.132 *χ*^2^ = 9.820
	Active	4 (4.76)	0	0	3 (14.28)	1 (4.76)	
	Latent	74 (88.09)	18 (85.71)	21 (100)	17 (80.95)	18 (85.71)	
Gluteus maximus	Does not have	17 (20.23)	5 (23.80)	9 (42.85)	2 (9.52)	1 (4.76)	*p* < 0.01 * χ^2^ = 19.280
	Active	7 (8.33)	0	1 (4.76)	1 (4.76)	5 (23.80)	
	Latent	60 (71.42)	16 (76.19)	11 (52.38)	18 (85.71)	15 (71.42)	
Gluteus medius	Does not have	9 (10.71)	4 (19.04)	3 (14.28)	0	2 (9.52)	*p* = 0.042 *χ*^2^ = 13.053
	Active	8 (9.52)	0	0	5 (23.80)	3 (14.28)	
	Latent	67 (79.76)	17 (80.95)	18 (85.71)	16 (76.19)	16 (76.19)	
Gluteus minimus	Does not have	13 (15.47)	5 (23.80)	5 (23.80)	2 (9.52)	1 (4.76)	*p* = 0.219 *χ*^2^ = 8.269
	Active	5 (5.95)	1 (4.76)	0	3 (14.28)	1 (4.76)	
	Latent	66 (78.57)	15 (71.42)	16 (76.19)	16 (76.19)	19 (90.47)	
Quadratus lumborum	Does not have	4 (4.76)	3 (14.28)	0	1 (4.76)	0	*p* = 0.067 *χ*^2^ = 11.762
	Active	24 (28.57)	3 (14.28)	4 (19.04)	9 (42.85)	8 (38.09)	
	Latent	56 (66.66)	15 (71.42)	17 (80.95)	11 (52.38)	13 (61.90)	
Piriformis	Does not have	4 (4.76)	1 (4.76)	1 (4.76)	0	2 (9.52)	*p* = 0.082 *χ*^2^ = 11.209
	Active	28 (33.33)	4 (19.04)	4 (19.04)	12 (57.14)	8 (38.09)	
	Latent	52 (61.90)	16 (76.19)	16 (76.19)	9 (42.85)	11 (52.38)	

Abbreviations: NDNC: non-dysmenorrhea non-contraceptive group; NDWC: non-dysmenorrhea with contraceptives group; WDNC: with dysmenorrhea non-contraceptives group; WDWC: with dysmenorrhea with contraceptives group. A *p* < 0.01 with a 99% confidence interval (**Bold**) was considered statistically significant. * One-way ANOVA was used.

**Table 6:** Presence of MTrPs in the pelvic floor muscles evaluated in the menstrual phase.

Muscles	Presence of MTrPs	Total(n = 84)	NDNC(n = 21)	NDWC(n = 21)	WDNC(n = 21)	WDWC(n = 21)	*p*-Value * χ^2^
		n (%)	n (%)	n (%)	n (%)	n (%)	
Ischiocavernosus	Does not have	27 (32.14)	10 (47.61)	12 (57.14)	2 (9.52)	3 (14.28)	*p* < 0.01 * χ^2^ = 20.680
	Active	7 (8.33)	0	0	3 (14.28)	4 (19.04)	
	Latent	50 (59.52)	11 (52.38)	9 (42.85)	16 (76.19)	14 (66.66)	
Bulbospongiosus	Does not have	39 (46.42)	13 (61.90)	13 (61.90)	8 (38.09)	5 (23.80)	*p* = 0.125 *χ*^2^ = 9.990
	Active	4 (4.76)	0	1 (4.76)	1 (4.76)	2 (9.52)	
	Latent	41 (48.80)	8 (38.09)	7 (33.33)	12 (57.14)	14 (66.66)	
Transverse perineal	Does not have	37 (44.04)	12 (57.14)	12 (57.14)	7 (33.33)	6 (28.57)	*p* = 0.043 *χ*^2^ = 12.972
	Active	5 (5.95)	0	0	1 (4.76)	4 (19.04)	
	Latent	42 (50)	9 (42.85)	9 (42.85)	13 (61.90)	11 (52.38)	
Puborectalis	Does not have	12 (14.28)	6 (28.57)	4 (19.04)	1 (4.76)	1 (4.76)	*p* = 0.268 *χ*^2^ = 7.610
	Active	28 (33.33)	5 (23.80)	6 (28.57)	9 (42.85)	8 (38.09)	
	Latent	44 (52.38)	10 (47.61)	11 (52.38)	11 (52.38)	12 (57.14)	
Pubococcygeus	Does not have	6 (7.14)	5 (23.80)	0	0	1 (4.76)	*p* = 0.001 * χ^2^ = 22.140
	Active	41 (48.80)	7 (33.33)	6 (28.57)	14 (66.66)	14 (66.66)	
	Latent	37 (44.04)	9 (42.85)	15 (71.42)	7 (33.33)	6 (28.57)	
Iliococcygeus	Does not have	1 (1.19)	1 (4.76)	0	0	0	*p* = 0.035 *χ*^2^ = 13.539
	Active	58 (69.04)	12 (57.14)	11 (52.38)	20 (95.23)	15 (71.42)	
	Latent	25 (29.76)	8 (38.09)	10 (47.61)	1 (4.76)	6 (28.57)	
Obturator internus	Does not have	0	0	0	0	0	*p* = 0.110 *χ*^2^ = 6.024
	Active	35 (41.66)	6 (28.57)	6 (28.57)	11 (52.38)	12 (57.14)	
	Latent	49 (58.33)	15 (71.42)	15 (71.42)	10 (47.61)	9 (42.85)	
Coccygeus	Does not have	32 (38.09)	9 (42.85)	8 (38.09)	8 (38.09)	7 (33.33)	*p* = 0.274 *χ*^2^ = 7.532
	Active	13 (15.47)	3 (14.28)	0	4 (19.04)	6 (28.57)	
	Latent	39 (46.42)	9 (42.85)	13 (61.90)	9 (42.85)	8 (38.09)	
Anal sphincter	Does not have	58 (69.04)	12 (57.14)	16 (76.19)	15 (71.42)	15 (71.42)	*p* = 0.774 *χ*^2^ = 3.268
	Active	5 (5.95)	1 (4.76)	1 (4.76)	2 (9.52)	1 (4.76)	
	Latent	21 (25)	8 (38.09)	4 (19.04)	4 (19.04)	5 (23.80)	

Abbreviations: NDNC: non-dysmenorrhea non-contraceptive group; NDWC: non-dysmenorrhea with contraceptives group; MTrP: myofascial trigger points; WDNC: with dysmenorrhea non-contraceptives group; WDWC: with dysmenorrhea with contraceptives group. A *p* < 0.01 (**Bold**) was considered statistically significant. * One-way ANOVA was used.

**Table 7:** Presence of MTrPs in the external muscles evaluated in the ovulatory phase.

Muscles	Presence of MTrPs	Total (n = 84)	NDNC (n = 21)	NDWC (n = 21)	WDNC (n = 21)	WDWC (n = 21)	*p*-Value * χ^2^
		n (%)	n (%)	n (%)	n (%)	n (%)	
Rectus abdominis	Does not have	13 (15.47)	6 (28.57)	3 (14.28)	1 (4.76)	3 (14.28)	*p* = 0.073 *χ*^2^ = 11.554
	Active	3 (3.57)	3 (14.28)	0	4 (19.04)	6 (28.57)	
	Latent	58 (69.04)	12 (57.14)	18 (85.71)	16 (76.19)	12 (57.14)	
External oblique	Does not have	20 (23.80)	7 (33.33)	5 (23.80)	3 (14.28)	5 (23.80)	*p* = 0.216 *χ*^2^ = 8.310
	Active	2 (2.38)	0	0	0	2 (9.52)	
	Latent	62 (73.80)	14 (66.66)	16 (76.19)	18 (85.71)	14 (66.66)	
Internal oblique	Does not have	55 (65.47)	17(80.95)	18 (85.71)	10 (47.61)	10 (47.61)	*p* = 0.021 *χ*^2^ = 14.959
	Active	10 (11.90)	0	2 (9.52)	3 (14.28)	5 (23.80)	
	Latent	19 (22.61)	4 (19.04)	1 (4.76)	8 (38.09)	6 (28.57)	
Adductor magnus	Does not have	14 (16.66)	5 (23.80)	5 (23.80)	0	4 (19.04)	*p* = 0.177 *χ*^2^ = 8.944
	Active	1 (1.19)	0	0	0	1 (4.76)	
	Latent	69 (82.14)	16 (76.19)	16 (76.19)	21 (100)	16 (76.19)	
Gluteus maximus	Does not have	19 (22.61)	6 (28.57)	7 (33.33)	3 (14.28)	3 (14.28)	*p* = 0.683 *χ*^2^ = 3.951
	Active	5 (5.95)	1 (4.76)	1 (4.76)	2 (9.52)	1 (4.76)	
	Latent	60 (71.42)	14 (66.66)	13 (61.90)	16 (76.19)	17(80.95)	
Gluteus medius	Does not have	20 (23.80)	4 (19.04)	9 (42.85)	3 (14.28)	4 (19.04)	*p* = 0.379 *χ*^2^ = 6.407
	Active	5 (5.95)	1 (4.76)	1 (4.76)	1 (4.76)	2 (9.52)	
	Latent	59 (70.23)	16 (76.19)	11 (52.38)	17 (80.95)	15 (71.42)	
Gluteus minimus	Does not have	16 (19.04)	5 (23.80)	6 (28.57)	2 (9.52)	3 (14.28)	*p* = 0.500 *χ*^2^ = 4.875
	Active	4 (4.76)	1 (4.76)	0	1 (4.76)	2 (9.52)	
	Latent	64 (76.19)	15 (71.42)	15 (71.42)	18 (85.71)	16 (76.19)	
Quadratus lumborum	Does not have	10 (11.90)	4 (19.04)	2 (9.52)	3 (14.28)	1 (4.76)	*p* = 0.082 *χ*^2^ = 11.209
	Active	15 (17.85)	4 (19.04)	1 (4.76)	2 (9.52)	8 (38.09)	
	Latent	59 (70.23)	13 (61.90)	18 (85.71)	16 (76.19)	12 (57.14)	
Piriformis	Does not have	17 (20.23)	5 (23.80)	3 (14.28)	7 (33.33)	2 (9.52)	*p* = 0.358 *χ*^2^ = 6.616
	Active	21 (25)	3 (14.28)	5 (23.80)	6 (28.57)	7 (33.33)	
	Latent	46 (54.76)	13 (61.90)	13 (61.90)	8 (38.09)	12 (57.14)	

Abbreviations: NDNC: non-dysmenorrhea non-contraceptive group; NDWC: non-dysmenorrhea with contraceptives group; WDNC: with dysmenorrhea non-contraceptives group; WDWC: with dysmenorrhea with contraceptives group. A *p* < 0.01 was considered statistically significant. * One-way ANOVA was used.

**Table 8:** Presence of MTrPs in the pelvic floor muscles evaluated in the ovulatory phase.

Muscles	Presence of MTrPs	Total (n = 84)	NDNC (n = 21)	NDWC (n = 21)	WDNC (n = 21)	WDWC (n = 21)	*p*-Value * χ^2^
		n (%)	n (%)	n (%)	n (%)	n (%)	
Ischiocavernosus	Does not have	39 (46.42)	14 (66.66)	14 (66.66)	7 (3333)	4 (19.04)	*p* < 0.01 * χ^2^ = 16.969
	Active	4 (4.76)	1 (4.76)	1 (4.76)	0	2 (9.52)	
	Latent	41 (48.80)	6 (28.57)	6 (28.57)	14 (66.66)	15 (71.42)	
Bulbospongiosus	Does not have	56 (66.66)	17 (80.95)	17 (80.95)	13 (61.90)	9 (42.85)	*p* = 0.052 *χ*^2^ = 12.476
	Active	1 (1.19)	0	0	1 (4.76)	0	
	Latent	27 (32.14)	4 (19.04)	4 (19.04)	7 (33.33)	12 (57.14)	
Transverse perineal	Does not have	52 (61.90)	13 (61.90)	16 (76.19)	11 (52.38)	12 (57.14)	*p* = 0.600 *χ*^2^ = 4.573
	Active	5 (5.95)	2 (9.52)	0	1 (4.76)	2 (9.52)	
	Latent	27 (32.14)	6 (28.57)	5 (23.80)	9 (42.85)	7 (33.33)	
Puborectalis	Does not have	18 (21.42)	4 (19.04)	10 (47.61)	1 (4.76)	3 (14.28)	*p* = 0.030 *χ*^2^ = 14.000
	Active	22 (26.19)	6 (28.57)	2 (9.52)	8 (38.09)	6 (28.57)	
	Latent	44 (52.38)	11 (52.38)	9 (42.85)	12 (57.14)	12 (57.14)	
Pubococcygeus	Does not have	21 (25)	6 (28.57)	8 (38.09)	6 (28.57)	1 (4.76)	*p* = 0.116 *χ*^2^ = 10.210
	Active	35 (41.66)	6 (28.57)	6 (28.57)	10 (47.61)	13 (61.90)	
	Latent	28 (33.33)	9 (42.85)	7 (33.33)	5 (23.80)	7 (33.33)	
Iliococcygeus	Does not have	15 (17.85)	6 (28.57)	8 (38.09)	1 (4.76)	0	*p* < 0.001 * χ^2^ = 25.286
	Active	51 (60.71)	7 (33.33)	8 (38.09)	18 (85.71)	18 (85.71)	
	Latent	18 (21.42)	8 (38.09)	5 (23.80)	2 (9.52)	3 (14.28)	
Obturator internus	Does not have	6 (7.14)	2 (9.52)	4 (19.04)	0	0	*p* = 0.049 *χ*^2^ = 12.659
	Active	28 (33.33)	4 (19.04)	5 (23.80)	8 (38.09)	11 (52.38)	
	Latent	50 (59.52)	15 (71.42)	12 (57.14)	13 (61.90)	10 (11.90)	
Coccygeus	Does not have	39 (46.42)	12 (57.14)	10 (47.61)	12 (57.14)	5 (23.80)	*p* = 0.55 *χ*^2^ = 12.314
	Active	7 (8.33)	0	1 (4.76)	1 (4.76)	5 (23.80)	
	Latent	38 (45.23)	9 (42.85)	10 (47.61)	8 (38.09)	11 (52.38)	
Anal sphincter	Does not have	69 (82.14)	17 (80.95)	21 (100)	17 (80.95)	14 (66.66)	*p* < 0.01 * χ^2^ = 18.435
	Active	5 (5.95)	3 (14.28)	0	2 (9.52)	0	
	Latent	10 (11.90)	1 (4.76)	0	2 (9.52)	7 (33.33)	

Abbreviations: NDNC: non-dysmenorrhea non-contraceptive group; NDWC: non-dysmenorrhea with contraceptives group; MTrPs: myofascial trigger points; WDNC: with dysmenorrhea non-contraceptives group; WDWC: with dysmenorrhea with contraceptives group. A *p* < 0.01 (**Bold**) was considered statistically significant. * One-way ANOVA was used.

**Table 9:** Pain reproduced at specific sites after palpation in 84 participants during menstrual phase.

	Self-Reported Pain Site																		
Muscles	Flank n (%)	Iliac Fossa n (%)	Hypogastrium n (%)	Mesogastrium n (%)	Epigastrium n (%)	Anterior Part Lower Limb n (%)	Posterior Part Lower Limb n (%)	Pubis n (%)	Groin n (%)	Gluteus n (%)	Lumbar n (%)	Coccyx n (%)	Sacrum n (%)	Internal Part Lower Limb n (%)	Vulva n (%)	Vagina n (%)	Urethra n (%)	Anus n (%)	Rectum n (%)
**Rectus Abdominis**	54 (64.29)	34 (40.48)	68 (80.95)	61 (72.62)	24 (28.57)	1 (1.19)	0 (0)	10 (11.90)	7 (8.33)	1 (1.19)	4 (4.76)	0 (0)	0 (0)	0 (0)	0 (0)	0 (0)	0 (0)	0 (0)	0 (0)
**External Oblique**	56 (66.67)	60 (71.43)	7 (8.33)	4 (4.76)	0 (0)	4 (4.76)	1 (1.19)	3 (3.57)	4 (4.76)	0 (0)	4 (4.76)	0 (0)	0 (0)	0 (0)	0 (0)	0 (0)	0 (0)	0 (0)	0 (0)
**Internal Oblique**	1 (1.19)	12 (14.29)	24 (28.57)	0 (0)	0 (0)	10 (11.90)	0 (0)	10 (11.90)	14 (16.67)	0 (0)	0 (0)	1 (1.19)	1 (1.19)	0 (0)	0 (0)	0 (0)	0 (0)	0 (0)	0 (0)
**Adductor Magnus**	0 (0)	0 (0)	1 (1.19)	0 (0)	0 (0)	7 (8.33)	3 (3.57)	2 (2.38)	2 (2.38)	0 (0)	0 (0)	1 (1.19)	0 (0)	75 (89.29)	0 (0)	0 (0)	0 (0)	0 (0)	0 (0)
**Gluteus Maximus**	0 (0)	1 (1.19)	0 (0)	0 (0)	0 (0)	5 (5.95)	1 (1.19)	0 (0)	0 (0)	64 (76.19)	12 (14.29)	3 (3.57)	4 (4.76)	9 (10.71)	0 (0)	0 (0)	0 (0)	0 (0)	0 (0)
**Gluteus Medius**	1 (1.19)	1 (1.19)	1 (1.19)	0 (0)	0 (0)	4 (4.76)	9 (10.71)	1 (1.19)	3 (3.57)	74 (88.10)	5 (5.95)	3 (3.57)	4 (4.76)	0 (0)	1 (1.19)	0 (0)	0 (0)	0 (0)	0 (0)
**Gluteus Minimus**	0 (0)	1 (1.19)	1 (1.19)	0 (0)	0 (0)	9 (10.71)	15 (17.86)	1 (1.19)	2 (2.38)	70 (83.33)	5 (5.95)	0 (0)	0 (0)	1 (1.19)	0 (0)	0 (0)	0 (0)	0 (0)	0 (0)
**Quadratus Lumborum**	3 (3.57)	3 (3.57)	5 (5.95)	1 (1.19)	1 (1.19)	1 (1.19)	2 (2.38)	2 (2.38)	2 (2.38)	20 (23.81)	75 (89.29)	1 (1.19)	8 (9.52)	0 (0)	0 (0)	0 (0)	0 (0)	0 (0)	0 (0)
**Piriformis**	1 (1.19)	7 (8.33)	11 (13.10)	1 (1.19)	0 (0)	6 (7.14)	10 (11.90)	10 (11.90)	17 (20.24)	48 (57.14)	6 (7.14)	11 (13.10)	9 (10.71)	2 (2.38)	16 (19.05)	3 (3.57)	2 (2.38)	8 (9.52)	0 (0)

**Table 10:** Pain reproduced at specific sites after palpation in 84 participants during ovulatory phase.

	Self-Reported Pain Site																		
Muscles n (%)	Flank n (%)	Iliac Fossa n (%)	Hypogastrium n (%)	Mesogastrium n (%)	Epigastrium n (%)	Anterior Part Lower Limb n (%)	Pubis n (%)	Groin n (%)	Vulva n (%)	Sacrum n (%)	Lumbar n (%)	Internal Part Lower Limb n (%)	Coccyx n (%)	Posterior Part Lower Limb n (%)	Gluteus n (%)	Vagina n (%)	Urethra n (%)	Anus n (%)	Rectum n (%)
**Rectus Abdominis**	15 (17.86)	25 (29.76)	56 (66.67)	50 (59.52)	14 (16.67)	1 (1.19)	1 (1.19)	4 (4.76)	1 (1.19)	1 (1.19)	5 (5.95)	0 (0)	0 (0)	0 (0)	0 (0)	0 (0)	0 (0)	0 (0)	0 (0)
**External Oblique**	43 (51.19)	56 (66.67)	5 (5.95)	2 (2.38)	2 (2.38)	5 (5.95)	0 (0)	1 (1.19)	0 (0)	1 (1.19)	2 (2.38)	0 (0)	0 (0)	0 (0)	0 (0)	0 (0)	0 (0)	0 (0)	0 (0)
**Internal Oblique**	0 (0)	10 (11.90)	11 (13.10)	0 (0)	0 (0)	4 (4.76)	6 (7.14)	4 (4.76)	0 (0)	0 (0)	0 (0)	1 (1.19)	0 (0)	0 (0)	0 (0)	0 (0)	0 (0)	0 (0)	0 (0)
**Adductor Magnus**	0 (0)	0 (0)	1 (1.19)	0 (0)	0 (0)	8 (9.52)	0 (0)	2 (2.38)	0 (0)	0 (0)	0 (0)	65 (77.38)	0 (0)	0 (0)	0 (0)	0 (0)	0 (0)	0 (0)	0 (0)
**Gluteus Maximus**	2 (2.38)	2 (2.38)	1 (1.19)	0 (0)	0 (0)	9 (10.71)	1 (1.19)	1 (1.19)	0 (0)	3 (3.57)	17 (20.24)	1 (1.19)	1 (1.19)	3 (3.57)	64 (76.19)	0 (0)	0 (0)	0 (0)	0 (0)
**Gluteus Medius**	1 (1.19)	1 (1.19)	0 (0)	0 (0)	0 (0)	6 (7.14)	1 (1.19)	1 (1.19)	0 (0)	1 (1.19)	6 (7.14)	0 (0)	0 (0)	7 (8.33)	61 (71.62)	0 (0)	0 (0)	0 (0)	0 (0)
**Gluteus Minimus**	0 (0)	1 (1.19)	2 (2.38)	0 (0)	0 (0)	8 (9.52)	0 (0)	0 (0)	0 (0)	2 (2.38)	9 (10.71)	1 (1.19)	1 (1.19)	7 (8.33)	62 (73.81)	0 (0)	0 (0)	0 (0)	0 (0)
**Quadratus Lumborum**	1 (1.19)	4 (4.76)	0 (0)	0 (0)	0 (0)	0 (0)	1 (1.19)	2 (2.38)	0 (0)	4 (4.76)	72 (85.71)	0 (0)	0 (0)	3 (3.57)	18 (21.43)	0 (0)	0 (0)	0 (0)	0 (0)
**Piriformis**	1 (1.19)	8 (9.52)	7 (8.33)	1 (1.19)	0 (0)	3 (3.57)	9 (10.71)	11 (13.10)	17 (20.24)	2 (2.38)	4 (4.76)	2 (2.38)	4 (4.76)	6 (7.14)	47 (55.95)	4 (4.76)	0 (0)	10 (11.90)	3 (3.57)

**Table 11:** Pain reproduced at specific sites after palpation in 84 participants during menstrual phase in the pelvic floor muscles.

	Self-Reported Pain Site																		
Muscles n (%)	Flank n (%)	Iliac Fossa n (%)	Hypogastrium n (%)	Mesogastrium n (%)	Epigastrium n (%)	Anterior Part Lower Limb n (%)	Posterior Part Lower Limb n (%)	Pubis n (%)	Groin n (%)	Gluteus n (%)	Lumbar n (%)	Coccyx n (%)	Sacrum n (%)	Internal Part Lower Limb n (%)	Vulva n (%)	Vagina n (%)	Urethra n (%)	Anus n (%)	Rectum n (%)
**Ischiocavernosus**	0 (0)	0 (0)	2 (2.38)	0 (0)	0 (0)	1 (1.19)	1 (1.19)	1 (1.19)	14 (16.67)	3 (3.57)	1 (1.19)	2 (2.38)	0 (0)	4 (4.76)	55 (65.48)	3 (3.57)	4 (4.76)	0 (0)	0 (0)
**Bulbospongiosus**	0 (0)	0 (0)	1 (1.19)	0 (0)	0 (0)	0 (0)	0 (0)	0 (0)	1 (1.19)	0 (0)	0 (0)	0 (0)	0 (0)	0 (0)	41 (48.81)	11 (13.10)	6 (7.14)	0 (0)	0 (0)
**Transverse Perineal**	0 (0)	0 (0)	0 (0)	0 (0)	0 (0)	1 (1.19)	1 (1.19)	0 (0)	1 (1.19)	21 (25.00)	0 (0)	2 (2.38)	0 (0)	2 (2.38)	29 (34.52)	1 (1.19)	0 (0)	9 (10.71)	0 (0)
**Puborectalis**	0 (0)	1 (1.19)	5 (5.95)	0 (0)	0 (0)	1 (1.19)	0 (0)	9 (10.71)	11 (13.10)	13 (15.48)	0 (0)	6 (7.14)	2 (2.38)	5 (5.95)	41 (48.81)	28 (33.33)	7 (8.33)	19 (22.62)	6 (7.14)
**Pubococcygeus**	0 (0)	13 (15.48)	23 (27.38)	0 (0)	0 (0)	5 (5.95)	9 (10.71)	14 (16.67)	18 (21.43)	14 (16.67)	4 (4.76)	5 (5.95)	2 (2.38)	6 (7.14)	31 (36.90)	14 (16.67)	4 (4.76)	6 (7.14)	0 (0)
**Iliococcygeus**	2 (2.38)	18 (21.43)	46 (54.76)	2 (2.38)	0 (0)	4 (4.76)	4 (4.76)	37 (44.05)	23 (27.38)	10 (11.90)	3 (3.57)	4 (4.76)	2 (2.38)	3 (3.57)	37 (44.05)	12 (14.29)	9 (10.71)	5 (5.95)	1 (1.19)
**Coccygeus**	0 (0)	2 (2.38)	3 (3.57)	0 (0)	0 (0)	0 (0)	2 (2.38)	1 (1.19)	3 (3.57)	20 (23.81)	2 (2.38)	21 (25.00)	5 (5.95)	1 (1.19)	13 (15.48)	2 (2.38)	0 (0)	20 (23.81)	6 (7.14)
**External Anal Sphincter**	0 (0)	0 (0)	0 (0)	0 (0)	0 (0)	0 (0)	0 (0)	0 (0)	0 (0)	4 (4.76)	0 (0)	1 (1.19)	0 (0)	0 (0)	7 (8.33)	0 (0)	0 (0)	26 (30.95)	0 (0)
**Obturator Internus**	0 (0)	7 (8.33)	17 (20.24)	0 (0)	0 (0)	9 (10.71)	33 (39.29)	19 (22.62)	19 (22.62)	42 (50.00)	9 (10.71)	8 (9.52)	8 (9.52)	13 (15.48)	35 (41.67)	10 (11.90)	6 (7.14)	25 (29.76)	7 (8.33)

**Table 12:** Pain reproduced at specific sites after palpation in 84 participants during ovulatory phase in the pelvic floor muscles.

	Self-Reported Pain Site																		
Muscles n (%)	Flank n (%)	Iliac Fossa n (%)	Hypogastrium n (%)	Mesogastrium n (%)	Epigastrium n (%)	Anterior Part Lower Limb n (%)	Pubis n (%)	Groin n (%)	Vulva n (%)	Sacrum n (%)	Lumbar n (%)	Internal Part Lower Limb n (%)	Coccyx n (%)	Posterior Part Lower Limb n (%)	Gluteus n (%)	Vagina n (%)	Urethra n (%)	Anus n (%)	Rectum n (%)
**Ischiocavernosus**	0 (0)	0 (0)	2 (2.38)	0 (0)	0 (0)	2 (2.38)	0 (0)	11 (13.10)	48 (57.14)	0 (0)	0 (0)	2 (2.38)	0 (0)	0 (0)	3 (3.57)	1 (1.19)	0 (0)	0 (0)	0 (0)
**Bulbospongiosus**	0 (0)	1 (1.19)	1 (1.19)	0 (0)	0 (0)	2 (2.38)	0 (0)	2 (2.38)	24 (28.57)	0 (0)	0 (0)	1 (1.19)	0 (0)	0 (0)	0 (0)	8 (9.52)	3 (3.57)	0 (0)	0 (0)
**Transverse Perineal**	0 (0)	0 (0)	0 (0)	0 (0)	0 (0)	1 (1.19)	0 (0)	2 (2.38)	18 (21.43)	1 (1.19)	1 (1.19)	0 (0)	0 (0)	0 (0)	16 (19.04)	2 (2.38)	1 (1.19)	7 (8.33)	0 (0)
**Puborectalis**	0 (0)	0 (0)	5 (5.95)	1 (1.19)	0 (0)	2 (2.38)	1 (1.19)	6 (7.14)	50 (59.52)	2 (2.38)	2 (2.38)	0 (0)	2 (2.38)	1 (1.19)	11 (13.10)	22 (26.19)	4 (4.76)	20 (23.81)	4 (4.76)
**Pubococcygeus**	1 (1.19)	2 (2.38)	22 (26.19)	0 (0)	0 (0)	4 (4.76)	18 (21.43)	16 (19.05)	23 (27.38)	1 (1.19)	2 (2.38)	6 (7.14)	3 (3.57)	5 (5.95)	10 (11.90)	11 (13.10)	6 (7.14)	6 (7.14)	0 (0)
**Iliococcygeus**	0 (0)	15 (17.86)	42 (50.00)	1 (1.19)	0 (0)	6 (7.14)	22 (26.19)	15 (17.86)	30 (35.71)	2 (2.38)	5 (5.95)	4 (4.76)	1 (1.19)	3 (3.57)	5 (5.95)	9 (10.71)	5 (5.95)	2 (2.38)	0 (0)
**Coccygeus**	1 (1.19)	2 (2.38)	3 (3.57)	0 (0)	0 (0)	3 (3.57)	3 (3.57)	3 (3.57)	9 (10.71)	4 (4.76)	0 (0)	1 (1.19)	12 (14.29)	0 (0)	13 (15.48)	1 (1.19)	0 (0)	19 (22.62)	5 (5.95)
**External Anal Sphincter**	0 (0)	0 (0)	0 (0)	0 (0)	0 (0)	0 (0)	0 (0)	0 (0)	3 (3.57)	2 (2.38)	0 (0)	0 (0)	1 (1.19)	0 (0)	4 (4.76)	0 (0)	0 (0)	18 (21.43)	0 (0)
**Obturator Internus**	2 (2.38)	10 (11.90)	17 (20.24)	1 (1.19)	0 (0)	6 (7.14)	11 (13.10)	14 (16.67)	33 (39.29)	3 (3.57)	4 (4.76)	8 (9.52)	4 (4.76)	23 (27.38)	38 (45.24)	12 (14.29)	4 (4.76)	23 (27.38)	5 (5.95)

## Data Availability

Raw data are available upon corresponding author request.
